# Repeated mass distributions and continuous distribution of long-lasting insecticidal nets: modelling sustainability of health benefits from mosquito nets, depending on case management

**DOI:** 10.1186/1475-2875-12-401

**Published:** 2013-11-07

**Authors:** Olivier JT Briët, Melissa A Penny

**Affiliations:** 1 Department of Epidemiology and Public Health, Swiss Tropical and Public Health Institute, Basel, Switzerland; 2 University of Basel, Basel, Switzerland

**Keywords:** Modelling, LLINs, Case management, Sustainability, Acquired immunity

## Abstract

**Background:**

Stagnating funds for malaria control have spurred interest in the question of how to sustain the gains of recent successes with long-lasting insecticidal nets (LLINs) and improved case management (CM). This simulation study examined the malaria transmission and disease dynamics in scenarios with sustained LLINs and CM interventions and tried to determine optimal LLIN distribution rates. The effects of abruptly halting LLIN distribution were also examined.

**Methods:**

Dynamic simulations of malaria in humans and mosquitoes were run on the OpenMalaria platform, using stochastic individual-based simulation models. LLINs were distributed in a range of transmission settings, with varying CM coverage levels.

**Results:**

In the short-term, LLINs were beneficial over the entire transmission spectrum, reducing both transmission and disease burden. In the long-term, repeated distributions sustainably reduced transmission in all settings. However, because of the resulting reduction in acquired immunity in the population, the malaria disease burden, after initially being reduced, gradually increased and eventually stabilized at a new level. This new level was higher than the pre-intervention level in previously high transmission settings, if there is a maximum disease burden in the relationship between transmission and disease burden at intermediate transmission levels. This result could lead one to conclude that sustained LLIN distribution might not be cost-effective in high transmission settings in the long term. However, improved CM rendered LLINs more cost-effective in higher transmission settings than in those without improved CM and the majority of the African population lives in areas where CM and LLINs are sustainably combined. The effects of changes in LLIN distribution rate on cost-effectiveness were relatively small compared to the effects of changes in transmission setting and CM. Abruptly halting LLIN distribution led to temporary morbidity peaks, which were particularly large in low to intermediate transmission settings.

**Conclusions:**

This study reaffirms the importance of context specific intervention planning. Intervention planning must include combinations of malaria vector control and CM, and must consider both the pre-intervention transmission level and the intervention history to account for the loss of immunity and the potential for rebounds in disease burden.

## Background

Sustaining the gains of recent malaria control successes, primarily achieved with long-lasting insecticidal nets (LLINs) and improved case management (CM), is attracting global interest. The current economic crisis has resulted in, at best, stagnating funds for malaria control [1]. However, disbursements are increasingly needed to compensate for reductions in acquired immunity —the result of transmission reductions caused by the large-scale roll-out of LLINs [2]. Rationally planning the most cost-effective allocation of malaria control resources is both urgent and complex.

Cost-effectiveness estimates of single malaria interventions and intervention packages [3-7] often assume fixed numbers of episodes and deaths averted per intervention unit and do not account for malaria transmission dynamics, or do so indirectly [8]. Dynamic models of malaria, such as those deployed on the OpenMalaria modelling platform [9], can capture the effects of reducing immunity in the population on the cost-effectiveness of intervention packages.

A previous analysis of LLINs in OpenMalaria [10] focused on the duration of the effective period (life time) of a single mass distribution of long-lasting insecticidal nets (LLINs) and studied its sensitivity to several parameters. The outcome variable analysed was the effective period from the time of distribution until the impact had reduced to half of its maximum. The reasoning was that at half impact, it would be wise to distribute more nets to compensate for the lost nets and sustain impact. That study [10] also showed that under a hypothetical situation with sustained LLIN coverage, without attrition or decay, the number of episodes would rebound (despite the sustained coverage) to a new equilibrium level. This was presumably due to reduced acquisition of immunity in the population. In high transmission situations, the new equilibrium level of disease burden was often higher than the burden before LLIN implementation, even though transmission was reduced. These results are consistent with the findings of Marsh and Snow [11], that in areas of high transmission, altering immunity profiles via interventions could lead to increased malaria burden if not accompanied with improved case management.

Building on the analysis of LLINs [10], this paper addresses four issues: firstly, the sustainability of health benefits from repeated mass distributions and continuous distribution of LLINs over longer time periods, taking attrition and decay into account; secondly, the sensitivity of the expected health benefits to transmission and distribution parameters; thirdly, how this interacts with the probability of treating malarial episodes, i.e. the case management (CM) level; finally, the potential consequences of halting LLIN distribution abruptly after an extended period of repeated mass distributions and continuous distributions.

## Methods

A simulation experiment was set up in OpenMalaria, similar to the one described previously by Briët and colleagues [10]. Briefly, the OpenMalaria modelling platform [9] is an open source programme in which stochastic individual-based models for malaria in humans are combined with a deterministic model for malaria in mosquitoes. Fourteen different model variants were used, each based on different assumptions [12]. Each simulated malaria infection has a distinct parasite density that varies by a five-day time step, while the malaria transmission level varies seasonally. In previous work [10], values of key parameters were varied around a “central scenario”, where 70% of a simulated population of 10,000 individuals received an LLIN that decayed over time. In this previous work, the main outcome of measuring impact was episodes averted. Here, in addition to events averted, the impact on both transmission and disability adjusted life years (DALYs) due to malaria is analysed.

In order to investigate the health benefits from repeated mass distributions and continuous distribution of LLINs in economic terms, this paper uses net health benefits (NHB) [13] as an economic outcome. The NHB are expressed in DALYs due to malaria, where malaria related deaths form by far the largest component. The costs and cost savings to the health system are also included in this statistic, using a ceiling ratio conversion factor of USD 235.28 ^2012^ per DALY. The method for calculating DALYs and NHB is described by Briët and colleagues [14]. In the literature, incremental cost-effectiveness ratios (ICERs) are often used to quantify cost-effectiveness of interventions such as LLINs [7], instead of NHB. ICERs have the disadvantage of being very large or very small when cases, deaths or DALYs averted are just above or below zero, respectively. Thus, they are not suitable for describing cost-effectiveness in situations where the effectiveness of interventions may be small, such as encountered here [see Additional file 1]. Impact on episodes, DALYs and NHB were calculated for both the whole population and for children less than five years of age.

For this experiment, the central scenario [described in Additional file 2] was identical to that used previously [10], except for the following differences:

i) The population size was 100,000 instead of 10,000 to account for stochasticity in rare events (deaths), necessary for DALY calculation.

ii) The accessible population proportion was set at 90% instead of 100%. This means that 10% of the population never received an LLIN, reflecting the reality that some people are out of reach of distribution campaigns for geographic or other reasons.

iii) Instead of a single mass distribution (five years into the simulation), mass distributions (to 70% of the accessible population [10]) were repeated every four years to recipients at random, irrespective of having (had) access to a net in a previous distribution.

iv) The simulation was extended to last 125 years

v) Eighty per cent of accessible new-borns and their mothers received access to (the use of) a new net through continuous distribution [15].

vi) The mosquito population had a susceptibility (to LLINs) profile as described [14] for the population ‘Pitoa’ to LLIN Permanet 2.0. This population showed 70% mortality in a 0.05% deltamethrin World Health Organization (WHO) cylinder / tube susceptibility test [16] and, in the absence of LLINs, 75% of mosquito-human contact occurred during times when people were sleeping indoors at night, while 25% occurred during other times [17]. Two assumptions were made: first, that mosquitoes repeated their host searching behaviour every feeding cycle [17] and second, that when someone already using an LLIN received a new one, the old one was discarded.

In addition to the changes listed above, several other outcome variables were monitored to calculate NHB. To analyse the sensitivity of NHB to changes in the model’s transmission and distribution parameters, the following variations of the central scenario were included:

i)  Fourteen model variants, each differing in their assumptions about malaria epidemiology [12];

ii)  Nine levels of pre-intervention entomological inoculation rates (EIR), at 1, 2, 4, 8, 16, 32, 64, 128 and 256 infectious bites per adult per annum (IBPAPA);

iii)  Mosquito insecticide susceptibility profiles of highly resistant (population ‘Akron’ [18]) and susceptible populations (population ‘Zeneti’ [19]), in addition to the intermediate ‘Pitoa’ population;

iv)  LLIN attrition half-lives of three and five years, in addition to the four years used in the central scenario;

v)  Distribution round intervals of three and five years, as well as the four years used in the central scenario;

vi)  Distribution round coverage levels of 60 and 80%, in addition to the 70% used in the central scenario, of the accessible population (90% of the total human population);

vii)  Continuous coverage levels at birth of 0%, 70%, 80% (central scenario) and 90%, as well as a double net distribution at 80%, approximating the effects of continuous net distribution both through antenatal campaigns (ANC) and through the Expanded Programme on Immunization (EPI).

viii)  For uncomplicated malaria episodes, five-day treatment probabilities of 0.05 [20,21] (baseline in the central scenario), 0.23712 and 0.4256. The five-day treatment probability of 0.05 corresponds to a level of 9% reported treatment of recalled fevers with an effective antimalarial drug in Demographic and Health Surveys (DHS) and Malaria Indicator Surveys (MIS) [22]. The five-day treatment probability of 0.2371 corresponds to a 2010 Tanzanian DHS level of reported treatment of recalled fevers of 59% [23]. The five-day treatment probability of 0.4256 corresponds to a level of reported treatment of recalled fevers of 80%. This is lower than Roll Back Malaria’s 2010 target of 80% of cases treated with effective anti-malarial medicines within one day of the onset of illness [24]. Due to recall bias, the true proportion of treated cases will be lower than the recalled proportion (fevers that are not treated are less often reported), and a proportion of fevers are not treated within one day of onset. Simulations used the baseline CM level corresponding to 9% treatment levels during the simulations’ warm up phase, and if scaled up, this was done automatically five years into the simulation. Health system costs were based on a fixed cost per treatment [25] and the cost of scaling up CM capacity (*e.g.* building extra health facilities) or behavioural change communication was not included. For severe malaria in hospital, the five-day treatment probability was kept constant at 48% [20].

ix)  LLIN distribution halted after 32 years (37 years into the simulation, after eight rounds of mass distributions every four years, supplemented with continuously distributed LLINs). This was done to assess how a sudden breakdown in LLIN distribution programmes would affect the malaria burden in areas where acquired immunity had been reduced substantially.

## Results and discussion

### Dynamics of malaria with continued interventions

**Figure 1:**
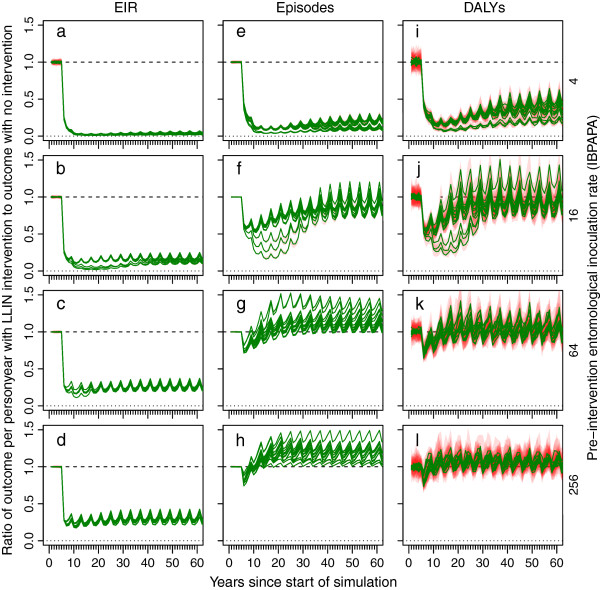
**Impact of LLINs on EIR, episodes and DALYs over time.** Green lines show medians and red polygons show ranges over 10 runs with unique seeds, of 14 model variants, of the ratio of the outcome variable in the intervention scenario relative to in the reference scenario (without intervention). **a – d)** entomological inoculation rate (EIR), **e – g)** episodes and **i – l)** disability adjusted life years (DALYs). Rows of panels have the same pre-intervention EIR.

Figure 1 shows the effects of distributing LLINs on malaria in the whole population (central scenario). These plots show the simulated EIR, episodes and DALYs relative to a non-intervention scenario for the first 60 years of the simulation, depending on pre-intervention EIR and model variant. All three outcomes declined sharply after LLIN distribution was initiated and rebounded to a new periodic stable state. The higher the pre-intervention transmission level, the faster the periodic stable state was reached. This is consistent with the observation in randomized controlled trials of insecticide treated nets (ITNs) that protective efficacy is reduced during the second year compared to the first year in areas of high transmission, but not in areas of low to moderate transmission [26]. For EIR, the new periodic stable state was only slightly higher than the lowest level reached (the median difference in the ratio of outcomes between the lowest level reached and the periodic stable state over all simulations was 0.04). However, the rebound was important for episodes (a median difference of 0.25) and DALYs (a median difference of 0.19) and varied with the pre-intervention transmission level. Versions of this figure and all following figures for outcomes calculated for children under five years of age only (instead of for the entire population) are presented in Additional file 3.

**Figure 2:**
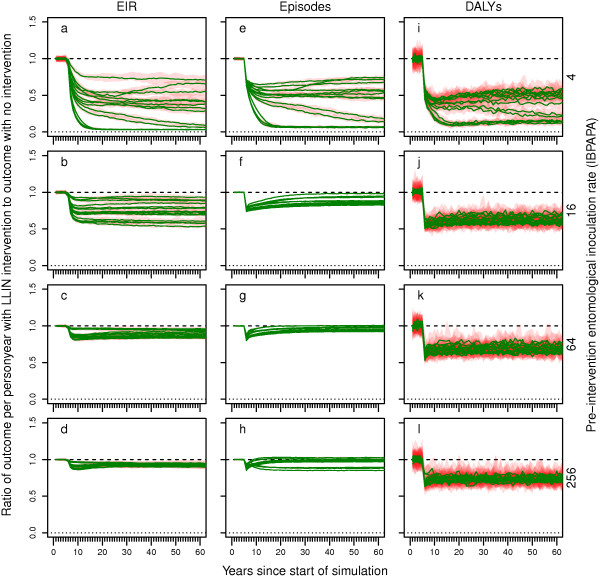
**Impact of scaled-up case management on EIR, episodes and DALYs over time. a – d)** entomological inoculation rate (EIR), **e – g)** episodes and **i – l)** disability adjusted life years (DALYs). Rows of panels have the same pre-intervention EIR.

The effects of scaling up CM to a level of 80% reported treatment of recalled fevers, compared to no intervention (with CM continued at a low level of 9% reported treatment of recalled fevers), are shown as ratios of outcomes (EIR, episodes, DALYs) per person year in Figure 2. These scenarios did not include LLIN distribution. At low and intermediate pre-intervention EIR levels, there was strong variability among the results from model variants, especially in terms of impact on the EIR. This variability is caused by differences among model variants in the relationship between prevalence of infectious people and EIR, depending on the level of access to care. Model variants R0063, R0065 and R0068 included heterogeneity in transmission; across CM levels, these model variants showed little variation in the proportion of bites on humans that are infectious to mosquitoes [see Additional file 4]. These model variants are represented by the highest curves in Figure 2a – d. In contrast, model variants R0115, R0125, R0132 and R0133 modelled quick decay of immunity; across CM levels, these model variants show a strong variation in the proportion of bites on humans that are infectious to mosquitoes. These model variants are represented by the lowest curves in Figure 2a – d. For a setting with a pre-intervention EIR of four IBPAPA, EIR, episodes and DALYs decreased strongly and then more or less plateaued, increased slightly or continued to decline, depending on the model variant. At higher pre-intervention EIR levels, EIR and episodes decreased slightly and slowly rebounded to a new level. DALYs, in contrast, decreased much more strongly and quickly, and then rebounded slightly but quickly to a new stable state. Scaled-up CM had some effect on transmission at lower pre-intervention EIR levels. However, it had little impact on transmission above an EIR of 64 IBPAPA. Similarly, its impact on the incidence of malaria episodes was negligible in such settings. The proportion of severe malaria disease and malaria related deaths that was averted by scaled-up management of uncomplicated malaria cases was considerable, even in high transmission settings (Note that treatment of severe malarial disease was not scaled-up in these scenarios and remained constant at 48%).

**Figure 3:**
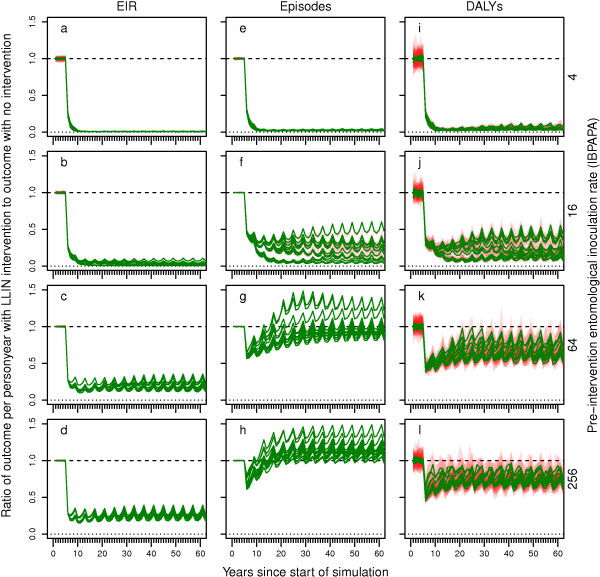
**Impact of LLIN distributions and scaled-up case management on EIR, episodes and DALYs over time. a – d)** entomological inoculation rate (EIR), **e – g)** episodes and **i – l)** disability adjusted life years (DALYs). Rows of panels have the same pre-intervention EIR.

The effects of both distributing LLINs and scaling up CM to achieve a level of 80% reported treatment of recalled fevers, compared to no intervention (apart from a low continued level of CM), are shown as ratios of outcomes (EIR, episodes, DALYs) per person year in Figure 3. For the EIR outcome (Figure 3a – 3d), these curves are similar to those shown in corresponding panels in Figure 1. For episodes (Figure 3e – 3h), the rebound was weaker in the scenarios with combined intervention than in the scenarios with LLIN distribution alone, especially in intermediate transmission settings (Figure 3f and 3g). For DALYs, at a pre-intervention EIR of 16 IBPAPA (Figure 3j), the rebound was much smaller with combined intervention than with either intervention alone. However, at higher pre-intervention EIRs (Figure 3k and 3l), the effect of the combined intervention was as follows: CM lowered the level and repeated LLIN distributions caused periodicity in the DALYs.

**Figure 4:**
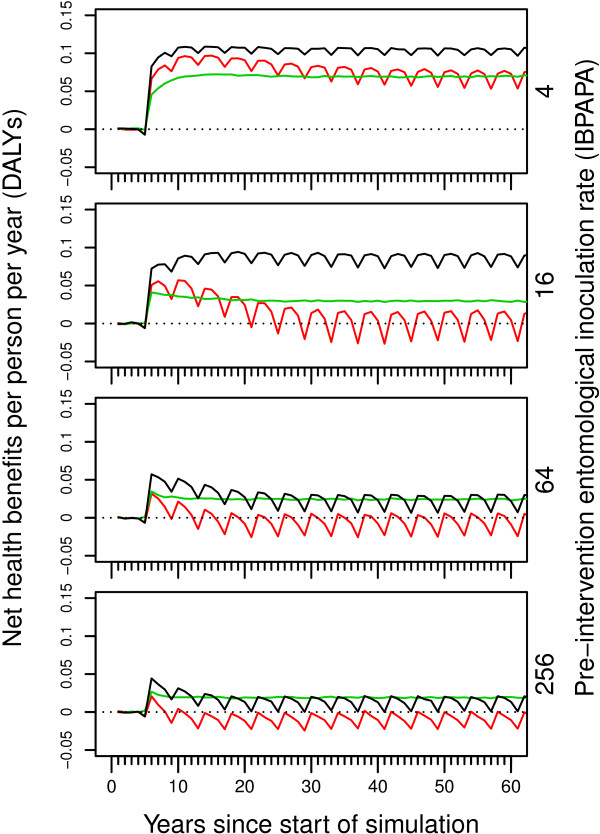
**Temporal dynamics of net health benefits of LLINs and case management.** Lines are goodness of fit-weighted averages of the 14 variants in the model ensemble. Net health benefits are calculated as compared to scenarios with a low baseline case management (CM) of 9% reported treatment of recalled fevers with an effective antimalarial drug in demographic health surveys or similar surveys. Red lines show the effect of only distributing long lasting insecticidal nets (LLINs). Green lines show the effect of only scaling up CM to 80% reported treatment of recalled fevers. Black lines show the effect of both distributing LLINs and scaling up CM to 80%.

Figure 4 shows the temporal dynamics of NHB of both LLINs and scaled-up CM expressed in DALYs, as compared to scenarios without scaled-up CM. In contrast to Figures 1, 2 and 3, where outcome variables are shown as a ratio of the results in the non-intervention scenarios, Figure 4 shows results in terms of an absolute difference. In Figures 1, 2 and 3, a curve is below the line where ‘ratio equals unity’ indicates a desired effect; in Figure 4, a curve above the horizontal line of ‘zero NHB’ indicates a desired effect. Also, instead of graphing all 14 model variants, a goodness of fit-weighted average of the model ensemble is plotted. The small dip in the fifth year after the start of the simulation (the red and black lines) is caused by the cost of the first round of LLIN distributions, which was incurred in the last 5-day period of the fifth year.

In the low transmission setting of four IBPAPA pre-intervention, the NHB of scaling up CM, of LLIN distribution and of combining the two interventions took five to 10 years to reach a maximum and declined slightly thereafter. In the transmission setting of 16 IBPAPA prior to intervention, the dynamic behaviour of the NHB of combined interventions was similar to that at four IBPAPA, but the NHB of individual interventions peaked earlier and declined steeply, particularly the NHB of LLINs. In transmission settings of 64 and 256 IBPAPA pre-intervention, the NHB of the combined intervention also peaked early and declined steeply. The NHB of LLINs alone were negative from the second round onward. NHB are presented cumulatively over time in Additional file 5.

**Figure 5:**
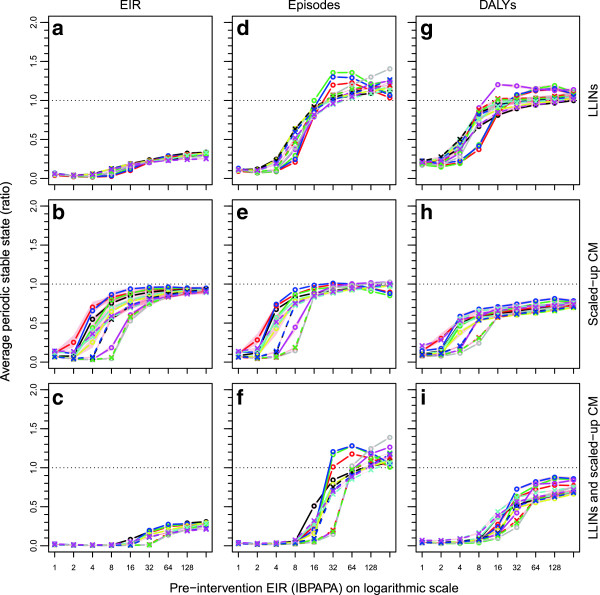
**Impact of LLINs, CM or both on EIR, episodes and DALYs at periodic stable state.** Ratios of results for intervention scenarios **(a**, **d** and **g**: long lasting insecticidal nets (LLINs); **b**, **e** and **h** scaled up case management (CM); and **c**, **f** and **i** both LLINs and scaled-up CM) and non-intervention scenarios (low CM only) calculated for means over the last 60 years of individual runs of 125 years, with 10 unique seeds per input EIR and model variant combination, for outcomes **a**, **b** and **c**: entomological inoculation rate (EIR): **d**, **e** and **f**: episodes; and **g**, **h** and **i**: disability adjusted life years (DALYs). Lines connect median values of groups of the ten seeds with the same input EIR and model variant. Model variants [12]: R0001 = solid black lines and circles; R0063 = solid red lines and circles; R0065 = solid lime green lines and circles; R0068 = solid blue lines and circles; R0111 = solid cyan lines and circles; R0115 = solid magenta lines and circles; R0121 = solid yellow lines and circles; R0125 = solid grey lines and circles; R0131 = dashed black lines and crosses; R0132 = dashed red lines and crosses; R0133 = dashed lime green lines and crosses; R0670 = dashed blue lines and crosses; R0674 = dashed cyan lines and crosses; R0678 = dashed magenta lines and crosses. Red polygons show ranges.

Figure 5 summarizes the results for EIR, episodes and DALYs as ratios of mean periodic stable states. These were calculated as averages over the last 60 years of the 125-year simulation runs. It was assumed that LLIN efficacy was constant over time (thus not affected by vector resistance to insecticides) and that there would be no further changes to the health system (including the vector control programme) or to socio-economic conditions. These assumptions may not be realistic, but the summary (at the stable state) is not subject to the transient dynamics occurring shortly after intervention at different rates depending on transmission setting. The relationships between outcomes with pre-intervention EIR were generally sigmoid, with inflection points between two and 32 IBPAPA, depending on model variant and outcome.

When LLINs were distributed to 63% of the human population in mass distributions and augmented with LLINs distributed to 72% of new-borns through neo-natal services, the EIR was strongly reduced in the low transmission settings. However, LLINs (only) reduced EIR by about 75% in the highest transmission setting (256 IBPAPA) considered (Figure 5a). This may seem low in comparison to, for instance, the 90% reduction observed in an ITN trial [27] with 72.3% user adherence [28], where annual EIR was estimated to be 237.25 – 288.35 IBPAPA, by Beier and colleagues [29]. An important difference in this simulation experiment is that mosquitoes were considered relatively resistant to pyrethoids (70% mortality in a 0.05% deltamethrin WHO susceptibility test [16]), whereas in the ITN trial, mosquitoes were fully susceptible. Furthermore, in the simulation experiment, 25% of the mosquitoes were assumed to be determined to bite during times when net users would not be under their nets [17]. With repeated long-term distributions, some resistance (behavioural, physiological or metabolic) is likely to develop and incorporating these settings in the central scenario may be more reasonable than assuming fully susceptible and vulnerable mosquitoes, with which these model variants simulate a much stronger reduction (than 90%) in EIR.

Below a pre-intervention EIR of 16 IBPAPA, the mean periodic stable state was below the initial levels for both number of episodes and DALYs (Figures 5d and 5g). However, for a pre-intervention EIR of 32 IBPAPA and above, the predicted mean number of episodes was higher than for non-intervention scenarios and the number of DALYs was higher than those in the non-intervention scenarios for some model variants (Figure 5g).

Although the stable states are useful for summarizing intervention effects, up to 60 years are required to reach such a state. Thus, stable states are generally not considered by malaria control programme managers because socio-economic developments and changes in the efficacy of malaria control tools make them unlikely. A shorter time horizon may be more relevant for public health decision making processes. Additional file 6: Figure S6.1 summarizes the results averaged over 13 – 16 years from the start of the simulation; that is, during the four years of the fourth LLIN distribution round. For this much shorter time horizon, the relationships are very similar except that the inflection points occur at somewhat higher pre-intervention EIRs.

**Figure 6:**
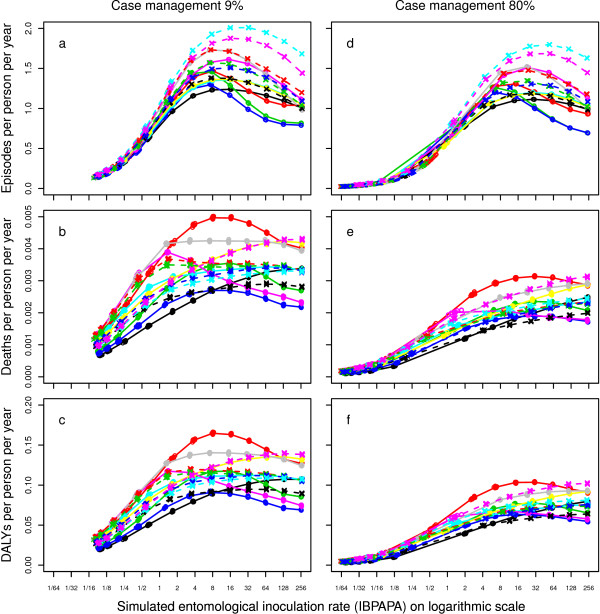
**Episodes, deaths, and DALYs depending on transmission and CM without LLINs.** Averages for the last 60 years of individual runs of 125 years, with 10 unique seeds per input EIR and model variant combination, with **a & d**) episodes per person per year **b & e**) direct and indirect deaths due to malaria, and **c & f**) disability adjusted life years (DALYs). Lines connect median values of groups of the ten seeds with the same input EIR and model variant. See the legend of Figure 5 for model variant [12] colour coding.

Under intervention in high transmission settings, the mean periodic stable state of the burden could be higher than the burden prior to intervention; this is a counter-intuitive result that that can be explained as follows: At equilibrium, the number of episodes in the population reaches a maximum at intermediate transmission levels. The relationship between transmission and malaria burden in these models^a^ is shown in Figure 6. The maximum in the number of episodes occurs around an EIR of 16 IBPAPA for most model variants [12] (Figure 6a and 6d). In a cross-sectional dataset comprising 10 sites, Marsh and Snow [11] show that severe malaria disease in children reaches a maximum at roughly 15% parasite prevalence. Their dataset was assembled prior to large scale LLIN intervention, thus it may be presumed that the sites included were also more or less in equilibrium at the time of measurement. Presenting cross-sectional data from Brazaville, Trape and Rogier [30] also show a similar peak in the relationship between burden and transmission. The observation of such maxima at intermediate transmission levels led the scientific community to speculate that as transmission reduces from high to intermediate levels, the malaria burden might increase. Ideally, such a hypothesis would be tested with longitudinal datasets in locations where increased vector control reduces transmission gradually from (very) high to intermediate levels, while CM coverage and effectiveness are constant. However, such locations are hard to find. Although vector control has increased since the year 2000 in most of sub-Saharan Africa, CM has changed as well, switching from chloroquine to sulfadoxine-pyrimethamine (SP) to artemisinin combination therapies (ACTs) as first line treatment, due to developing drug resistance. For instance, O’Meara and colleagues [31] did not observe an increasing malaria burden as parasite prevalence fell in Kilifi district, Kenya over the period 1990 – 2007, but the reduction in parasite prevalence after 1998 could be due to the switch to SP that year. The EIR in the area was, on average, 19.3 IBPAPA in 1997–1998 [32], although with strong spatial variation and thus perhaps too low to expect an increase in disease along with decreasing exposure.

A possible explanation for why transmission and malaria burden peak at intermediate EIR levels is that at (very) high EIR levels, people are quickly immunized and protected against severe disease by on-going immunity-maintaining infections. In these models, simulations with a weaker seasonality showed the peak number of episodes occurring at a lower EIR, probably because the immunity-maintaining infections are more evenly spread throughout the year (Stuckey, unpublished observations). However, there is some controversy over whether or not this phenomenon of a peak burden at intermediate EIR really exists. Problems with using cross-sectional data, due to confounders such as seasonality and innate immunity, have been pointed out [33-37]. The dataset of Marsh and Snow [11] was used, among others, to fit these models [38]. In order to investigate the sensitivity of this phenomenon to this cross sectional dataset, one model variant was refitted excluding the Marsh and Snow dataset [11]. Without the Marsh and Snow dataset [11], the relationship between EIR and total number of episodes was similar to that with the data set included [Additional file 7]. The relationship between EIR and DALYs was also similar but somewhat steeper than with the data included and the impact of LLINs still had negative NHB at high pre-intervention EIRs. Apparently, fitting these dynamic malaria models to data on ‘prevalence with age’ alone results in similar dynamics. This was also demonstrated in another malaria model [39,40].

At high transmission intensity, malarial disease is mostly concentrated among the youngest age groups. As transmission intensity decreases, malarial disease concentration shifts from the youngest age groups and spreads more evenly over all age groups, possibly leading to relatively more cerebral malaria and higher case fatalities at intermediate transmission levels [11,30]. Many malariological studies limit themselves to examining malaria in children under ten [11] or under five years of age [37], partly because the duration of immunity is poorly understood and because the high malaria burden in children makes this group one of special interest to malaria control programme planners. For this reason, the graphical analyses in this manuscript were repeated for children under five years of age only [see Additional file 3]. However, analyses for the whole population are preferred as the analyses for children under five do not capture the shifts of morbidity and mortality to older age groups and because DALYs already give deaths occurring at young ages more weight as a result of more years of life lost (YLLs).

The similarity between Figure 5b and 5e illustrates that, in scenarios with scaled-up CM as a single intervention, most of the reduction in episodes in low transmission settings was due to sustainably reduced transmission. At higher transmission settings, the reduction in absolute DALYs was due to fewer uncomplicated cases progressing into severe disease and death. The combination of both LLIN distribution and scaled-up CM reduced EIR more than LLIN distribution alone could achieve in pre-intervention transmission settings with between eight and 16 IBPAPA (Figure 5c), but did not reduce EIR much in higher transmission settings beyond 32 IBPAPA. This explains the curves in Figure 5f in comparison to Figure 5d. Similarly, this joint effect on transmission is one of the factors that explain the difference between Figure 5i and Figure 5g at intermediate pre-intervention EIR level. The general reduction in intermediate and high pre-intervention EIR settings due to scaled-up CM (Figure 5h) is another explanatory factor. Further, the difference in how the combined intervention affects DALYs as compared to LLIN intervention alone may be because of the altered relationship between transmission and disease burden due to CM. Panels d, e and f in Figure 6 show how disease burden relates to transmission at the higher CM level in the OpenMalaria models. For episodes, the plots are very similar to those from the low CM level situation. For deaths and DALYs, however, maxima at intermediate transmission levels were somewhat less pronounced at the high CM level compared to at the low CM level and the point at which malaria burden is highest shifted to a higher transmission level, if it occurred at all. CM thus alters the relationship between transmission and severe disease and allows transmission reducing LLINs to be more effective in decreasing malaria burden.

**Figure 7:**
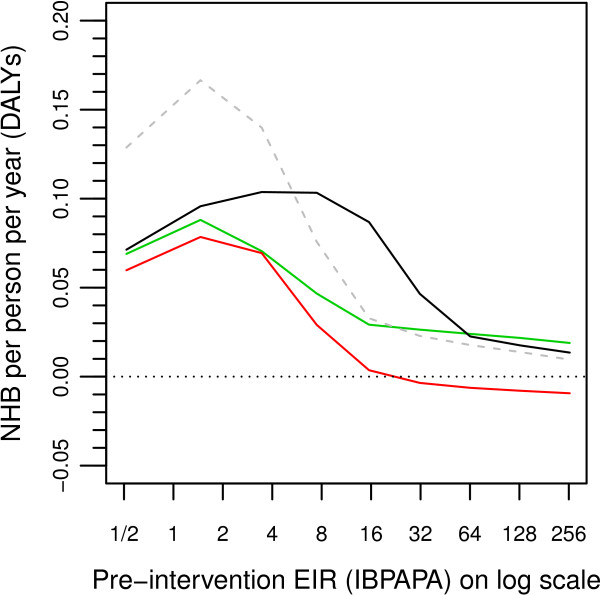
**Mean NHBs of LLINs and CM at periodic stable state.** Lines are goodness of fit-weighted averages of the 14-variant model ensemble, averaged over the last 60 years of the 125 year simulation period, where the net health benefits (NHB) are in a periodic stable state. NHB are calculated as compared to scenarios with a low baseline case management (CM) of 9% reported treatment of recalled fevers with an effective antimalarial drug in DHS type surveys. The red line shows the effect of only distributing LLINs. The green line shows the effect of only scaling up CM to 80% reported treatment of recalled fevers. The black line shows the effect of both distributing LLINs and scaling up CM to 80%. The grey dashed line is the sum of the red and green line.

Figure 7 allows for a direct comparison of the effects of individual (sustained LLIN distribution or scaled-up CM) and combined interventions, by showing the mean NHB of these interventions for the periodic stable state (the last 60 years of the 125-year simulation period) together in a single plot^b^. In low pre-intervention EIR settings, both LLINs and CM each had high NHB, but in combination they could not achieve more than what each intervention could achieve by itself because, in these low settings, malaria was prevented largely by either intervention. The NHB of either intervention was higher at a pre-intervention EIR of two IBPAPA than at one IBPAPA because there is more malaria to avert at the higher transmission setting. For the combined intervention, NHB continued to increase with increasing pre-intervention EIR, up to eight IBPAPA. However, for individual interventions, NHB declined in pre-intervention transmission settings above two IBPAPA, even though the preventable disease burden increased with increasing transmission up to about eight IBPAPA (Figure 6c). Thus, the decline in NHB for individual interventions between pre-intervention transmission settings of two and eight IBPAPA is presumably due to the increase in residual transmission (Figures 5a and 5b), which only becomes important for the combined intervention above eight IBPAPA (Figure 5c). The increase in residual transmission was strongest between four and 16 IBPAPA for individual interventions (Figures 5a and 5b) and between 16 and 64 IBPAPA for the combined intervention (Figure 5c), reflected in the steep declines in NHB with increasing pre-intervention EIR over these intervals (Figure 7). As a result, in intermediate transmission settings (*e.g.* an EIR of 16 IBPAPA), the combined intervention achieved much higher NHB than the sum of the effects of each individual intervention. The altered shape of the relationship between transmission and disease burden due to scaled-up CM plays only a minor role. Above an EIR of 64 IBPAPA, the combination was less cost-effective than CM alone. This is because above 64 IBPAPA, the average of model variants predicted that the burden would increase as EIR decreases at this high level of CM (Figure 6f).

Additional file 6: Figure S6.2 summarizes the NHB of the fourth LLIN distribution round. With a much a shorter time horizon, for an LLIN single intervention, the curve is slightly higher and shifted to the right, breaking the ‘zero NHB’ line at 32 IBPAPA instead of at 24 IBPAPA as at the stable state. For scaled-up CM, the relationship is very similar and for the combined intervention, the curve is nearly identical to the curve at the stable state.

**Figure 8:**
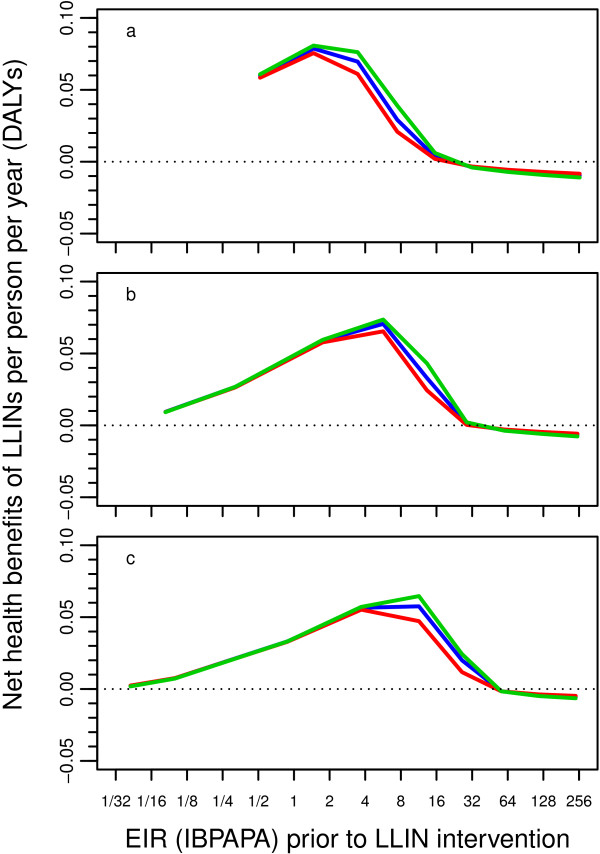
**NHBs of LLINs depending on CM and distribution rate.** Lines are goodness of fit-weighted averages of the 14 variant model ensemble, averaged over the last 60 years of the 125 year simulations. Green, blue and red lines represent scenarios where mass distributions were done every three, four and five years, respectively. **a**) Case management (CM) level of 9% reported treatment of recalled fevers with an effective antimalarial drug DHS type surveys. **b**) CM level of 59%. **c**) CM level of 80%.

Figure 8 shows the mean NHB of LLINs, depending on the transmission level^c^ prior to LLIN intervention and on distribution rate (mass distributions at intervals of three, four or five years) at the periodic stable state, for three levels of CM. With increasing CM levels, the maxima in the curves for NHB of LLINs shift to higher transmission levels and diminish slightly. In a situation with a low CM level, NHB of LLINs were negative above about 20 IBPAPA. With a high CM level, this was above about 56 IBPAPA. It should be noted that NHB were predicted to be negative for LLINs at higher pre-intervention EIR because DALYs themselves were negative, not because the costs were high. This result was relatively insensitive to the choice of ceiling ratio at USD 235.28 per DALY. With a higher ceiling ratio of, for example USD 1000 per DALY, the values would still have been negative. With a lower ceiling ratio (*e.g.* USD 100 per DALY), the values would have been more negative.

In settings with an intermediate EIR level prior to LLIN intervention, a higher distribution frequency was predicted to be more cost-effective. However, in the highest transmission settings, where predicted NHB of LLINs were negative, increasing the distribution rate made LLINs slightly less cost-effective. Also, with the highest CM level, in the lowest transmission setting simulated (less than 0.0625 IBPAPA), a higher distribution frequency was marginally less cost-effective. At this high CM level of 80% reported treatment of recalled fevers, varying distribution intervals (between three and five years) has surprisingly little impact on cost-effectiveness of LLINs for settings between one eighth and four IBPAPA prior to LLIN intervention, the extra costs being balanced out by slightly higher burden reductions.

In addition to examining the sensitivity of NHB of LLINs to transmission setting, CM level and LLIN mass distribution frequency, the sensitivity of NHB to mosquito susceptibility to pyrethroids, LLIN attrition half-life, population coverage of LLIN mass distributions and target population coverage in continuous distribution programmes were also studied. Additional file 8 contains the results of this sensitivity analysis for individual model variants around the central scenario, expressed in DALYs, with CM kept at the low baseline. Additional file 8: Figure S8.1a is comparable to the red line for LLINs in Figure 7, except that it shows results for each model variant separately, rather than a weighted average. At a pre-intervention EIR of 16 IBPAPA [Additional file 8: Figure S8.1], all model variants but four (R0115, R0125, R0132, R0133) predicted positive NHB for the central scenario. For transmission levels below eight IBPAPA, all model variants predicted positive health benefits, whereas for transmission levels above 32 IBPAPA, most variants predicted negative NHB.

The NHB of LLINs were very sensitive to the susceptibility of the mosquito population to pyrethroids [Additional file 8: Figure S8.1b] (although less sensitive than to the pre-intervention EIR), illustrating the threat of insecticide resistance to the sustainability of health benefits from LLIN distributions. The NHB were relatively insensitive to variations in attrition half-life [Additional file 8: Figure S8.1c], population coverage of LLIN mass distributions [Additional file 8: Figure S8.1e] and target population coverage of continuous distribution programmes [Additional file 8: Figure S8.1f], as well as to variations in mass distribution round intervals [Additional file 8: Figure S8.1d]. At a pre-intervention EIR of four IBPAPA [Additional file 8: Figure S8.2], where LLINs showed high cost-effectiveness for all model variants, sensitivity to pyrethroid susceptibility and LLIN distribution parameters was similar to the sensitivity at a pre-intervention EIR of 16 IBPAPA.

In general, where LLINs had positive NHB, NHB improved slightly with increasing coverage and distribution frequency for the ranges studied, despite the higher cost associated with higher LLIN distribution rates. This is because the differences in costs, expressed in DALYs, were insignificant compared to the difference in DALYs averted. For instance, the difference in cost between five-year round intervals of three-year intervals was a mere USD 0.28 per person per year, equivalent to 0.00176 DALYs at a ceiling ratio of USD 235.28 per DALY. This cost difference is further reduced by health systems cost savings due to fewer treatments at higher distribution rates.

The model variants showed large variations in the predicted effects of LLINs on the mean periodic stable state burden levels (Figure 5), as well as in sensitivities of LLIN NHB to aspects of the vector population, LLIN quality and distribution rate [Additional file 8: Figures S8.1 and S8.2]. These observations can be linked to strong variations in the impact of LLINs on transmission (Figure 5a, [see also Additional file 9]) and to the shape of the relationships between deaths, DALYs and transmission (Figure 6b and 6c). In Figure 6, some model variants (R0063, R0065, R0068) show a sharp peak between eight and 16 IBPAPA. One variant (R0115) shows a sharp peak around one IBPAPA, but for the other 10 variants, the relationships are increasing or relatively flat moving from low to high transmission. The strong differences among model variants in the relationship between transmission and disease burden reflect uncertainty about this relationship; the ensemble of variants encompasses a range of assumptions about malaria epidemiology and these were individually fit to malariological data sets. This dubiousness translates into uncertainty about the sensitivity of NHB to intervention scenarios.

### Dynamics of malaria after halting LLIN distribution

**Figure 9:**
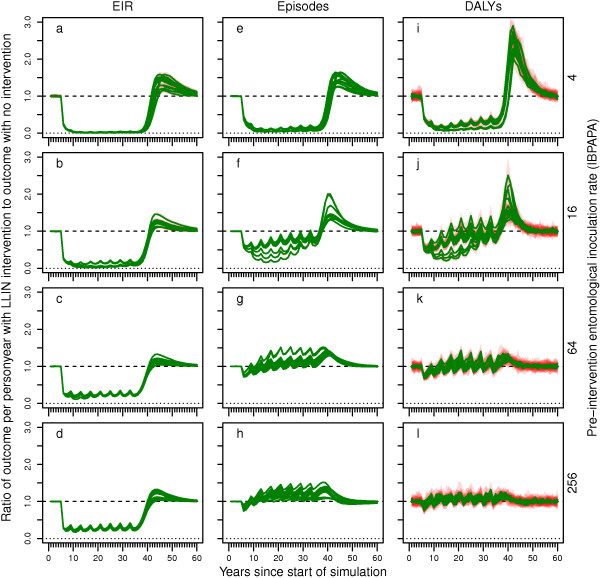
**Impact of halting LLIN distribution on EIR, episodes and DALYs for a central scenario over time. a – d)** entomological inoculation rate (EIR), **e – g)** episodes and **i – l)** disability adjusted life years (DALYs). Rows of panels have the same pre-intervention EIR.

**Figure 10:**
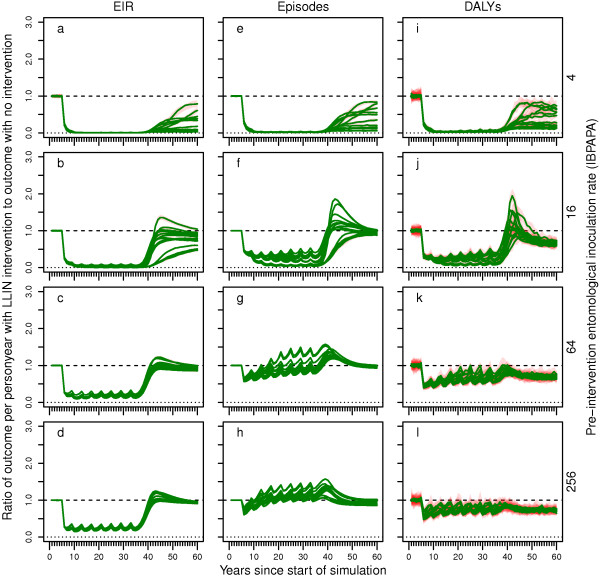
**Impact of halting LLIN distribution on EIR, episodes and DALYs with continued scaled-up CM. a – d)** entomological inoculation rate (EIR), **e – g)** episodes and **i – l)** disability adjusted life years (DALYs). Rows of panels have the same pre-intervention EIR.

**Figure 11:**
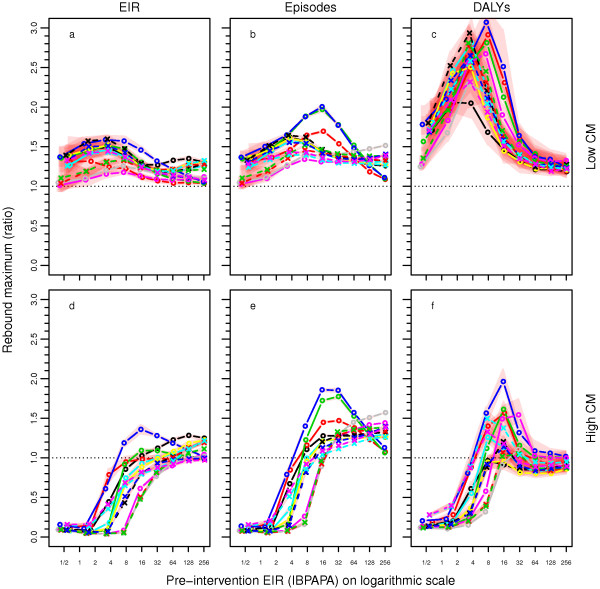
**Maximum rebound after abruptly halting LLIN distributions, depending on CM and pre-intervention EIR.** Maxima in the ratios of results after halting LLIN distribution after eight rounds (with **a**, **b** and **c**: low baseline CM and **d**, **e** and **f** scaled-up CM); and non-intervention scenarios (low CM only) calculated for means over the last 60 years of individual runs of 125 years, with 10 unique seeds per input EIR and model variant combination, for outcomes **a** and **d:** entomological inoculation rate (EIR): **b**, and **e**: episodes; and **c** and **f**: disability adjusted life years (DALYs). Lines connect median values of groups of the ten seeds with the same input EIR and model variant. See the legend of Figure 5 for model variant [12] colour coding. Red polygons show ranges. Note that if the rebound maximum is shown is below 1.0, it is possible that the maximum is not reached within 23 years post halting of LLIN distribution, and it is likely that in that case, the maximum is not larger than 1.0.

In a subset of scenarios, LLIN distribution was halted abruptly after 32 years of distribution (in eight rounds at four-year intervals, as well as continuously through ante-natal services). In some of these scenarios, CM was scaled up five years into the simulation and kept constant thereafter (even if LLIN distribution was halted). Figures 9 and 10 show the simulated malaria dynamics over time for a setting with low CM and scaled-up CM, respectively. After halting LLIN distribution (the last LLINs were distributed 37 years into the simulation), malaria increased rapidly to a level above the pre-intervention level and then declined slowly to the pre-intervention level. The maxima of these ‘rebounds’ were particularly high in the lower pre-intervention transmission settings. For instance, in a setting with a pre-intervention EIR of four IBPAPA, up to three times as many DALYs as in the pre-intervention stage were observed temporarily (Figure 9i and Figure 11c). In settings with 64 or more IBPAPA pre-intervention, the rebounds were relatively small, particularly for episodes and DALYs. However, a comparison between Figure 5g and Figure 11c shows that there is no linear relationship between the magnitude of the long-term reduction and the rebound maximum: the rebound maximum for DALYs was highest at pre-intervention EIRs of between four and eight IBPAPA, depending on the model variant, whereas the relative long-term reduction in malaria DALYs was intermediate in these transmission settings.

In situations where CM was scaled up at the same time as the first LLIN distribution and where CM was maintained at a high level, rebounds to above pre-intervention levels did not always occur (Figure 11d – f). For instance, in a setting with a pre-intervention EIR of below four IBPAPA, malaria increased gradually over time until it reached a new stable state, which was below the pre-intervention level (Figure 10). In settings with 64 or more IBPAPA pre-intervention, EIR and malaria episode dynamics were very similar to those for situations without scaled-up CM, although scaled-up CM maintained a lower level of DALYs (Figures 9 and 10). At intermediate pre-intervention EIR settings between four and 64 IBPAPA, with continued scaled-up CM, rebound maxima after halting LLIN distribution were still present, though mitigated and the size of these maxima varied strongly depending on the model variant. Note that in Figure 11d – f, the rebound maximum ratios are calculated in comparison to the non-intervention scenario with low CM. Thus, the rebound maximum ratios resulting from halting LLIN distribution, compared to scenarios with continued high CM (with lower malaria burden), would be stronger [see Additional file 10].

### Relative importance of transmission settings

**Figure 12:**
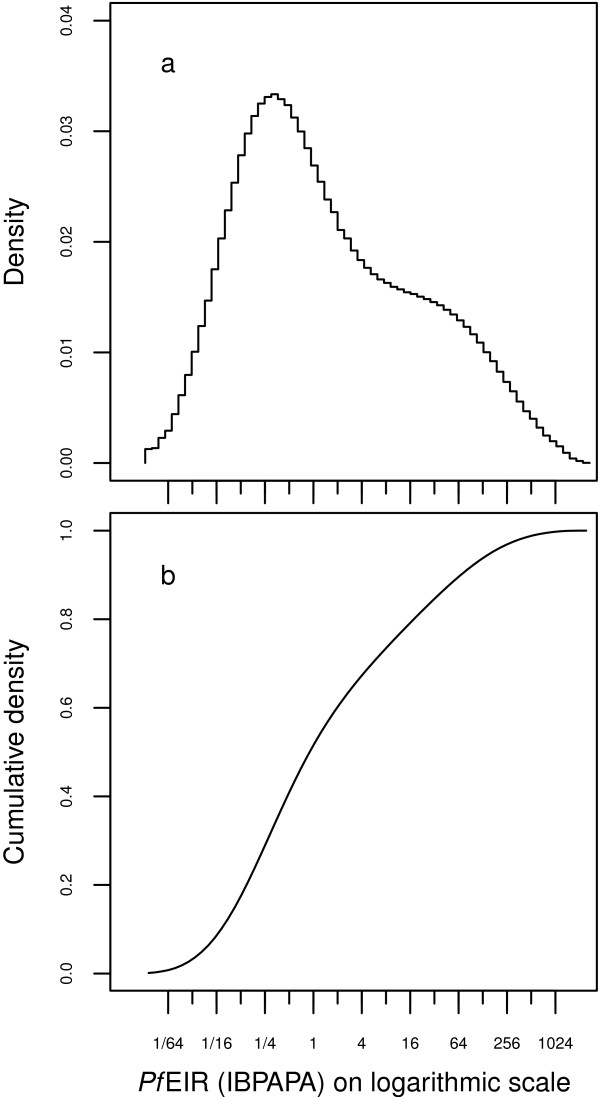
**Distribution of the African population over the transmission spectrum as of 2010. a)** Density and **b)** cumulative density. The distribution is calculated from the ‘*Pf*EIR’ map published by Gething and colleagues [41].

The 2010 distribution of the African population living in malaria endemic zones over the range of *Plasmodium falciparum* transmissions is shown in Figure 12. Such a figure could be helpful to quantify the prevalence of the different transmission settings in Africa and, thus, to assess the importance of the transmission-setting dependent findings. It should be noted that a pre-intervention distribution would be better suited to this study, since in 2010, the strong reduction in transmission in many African countries was likely due to the scaling up of LLINs. A pre-intervention distribution would probably be similar in shape to Figure 12, but be shifted towards higher transmission. At the time of writing, the Malaria Atlas Project is planning to produce a ‘pre-intervention’ transmission *P. falciparum* map that would exclude data points where malaria is likely affected by interventions (Gething, personal communication).

By comparing Figures 7 and 11 to Figure 12 (and assuming that the shift would be negligible), the following could be predicted: for a large proportion of the African population (about 60%) residing in areas with a lower range of transmission below about two IBPAPA, either sustained high levels of CM or sustained LLIN distributions would be cost-effective; a combination of the two interventions would have only marginally more NHB. However, a morbidity rebound (apparent as an epidemic) is unlikely to occur if LLIN distribution is halted while CM continues. For an additional 30% of the population, exposed to higher transmission up to about 64 IBPAPA, LLINs must be combined with CM in order to be most cost-effective, due to a synergistic effect. For this segment of the population, halting LLIN distribution abruptly (after a period of implementation) even while continuing high level CM could result in major epidemics, with peaks in episodes and DALYs up to two times those in the pre-intervention level. For the remaining 10% of the population, exposed to high transmission, scaling up and sustaining a high level of CM would be the best strategy for reducing the disease burden, as LLINs would not increase NHB. Somewhat surprisingly, halting LLIN distributions abruptly while continuing CM may not result in major epidemics in this segment of the population.

## Conclusions

Because of increasing concerns about how to sustain the gains of recent malaria control successes with LLINs and CM, this study simulated malaria in scenarios with sustained or abruptly halted LLIN distributions, with or without scaled-up CM. The modelling analysis confirmed that in the short term, LLIN distributions are very beneficial over the entire spectrum of transmission settings, reducing both transmission and disease burden. However, the longer-term dynamics indicated that, under sustained vector control, transmission and burden rebound to higher levels than those initially reached, with the level of the periodic stable state and the time needed to reach that stable state depending on the pre-intervention level of transmission.

In low to medium pre-intervention transmission settings (up to about 16 IBPAPA), immunity acquisition was reduced in simulation scenarios with sustained LLIN distribution and, as a result, malaria transmission and malaria disease burden increased over several decades until new stable states were reached, below the level prior to intervention. This increase happened while pyrethroid susceptibility of vector mosquitoes was considered constant. With evidence of building pyrethroid resistance [42] and high sensitivity of LLIN effectiveness to pyrethroid susceptibility in the simulation results, the estimated increases (rebounds) in disease burden are probably conservative.

In higher transmission settings, sustained distribution of LLINs resulted in transmission levels that stabilized at levels lower than those at pre-intervention. Whereas the new transmission level was lower than at pre-intervention, simulation results predicted that the disease burden could potentially be above pre-intervention levels in previously high transmission settings if transmission was not reduced sufficiently. As discussed by Marsh and Snow [11], the non-linear relationship between transmission and burden dictates that if transmission is decreased from a high to a medium level (but not much further), the burden might increase in the long term as immunity acquisition is reduced. To mitigate the potential for increased disease burden, Marsh and Snow [11] propose increasing the focus on CM. Some scientists may doubt the validity of Marsh and Snow’s [11] data and may argue that using their data to parameterize the models may force the observed negative NHB of LLINs at high EIRs. However, OpenMalaria models and other models [39,40] that did not include this dataset showed similar results; this is likely due to fitting the models to data on the relationship of parasite prevalence or parasite density with age.

Analysing the level of the stable state revealed a strong interaction between CM and LLINs on NHB. The direction of this interaction depended on the pre-intervention transmission setting. The interaction can be explained by both joint effects on transmission (reducing transmission below the intermediate transmission level) and CM altering the relationship between transmission and burden. Improved CM coverage rendered LLINs more cost-effective at higher transmission settings than without improved CM coverage. This indicates that in areas with medium transmission intensity, integrated control [11,43] will be highly cost-effective. However, in settings with a (very) high pre-intervention transmission level (roughly^d^, above 64 IBPAPA), the combination of both LLINs and improved CM was less cost-effective than CM alone in scenarios with sustained (very) high CM combined with sustained LLIN distribution and with no changes in LLIN efficacy nor in the malaria control programme. Thus, the results indicate that with a long-term perspective, distributing LLINs might not be cost-effective in areas [44] with such high transmission. This conclusion should be balanced with the knowledge that 90% of the population in African most likely lives in areas with much lower (potential) transmission.

The effects of changes in LLIN distribution rates were relatively small compared to the effects of transmission setting. The magnitude of the effects, however, was sensitive to transmission setting. Planning for optimal combinations of malaria control tools will need to take into account both the pre-intervention potential for transmission and existing CM levels and will thus need to be geographically specific. However, there is considerable uncertainty about these levels and a lack of information on the (probably) considerable short-range spatial heterogeneity, limiting the meaningful spatial resolution of such plans.

Simulation results indicate that after a period of vector control, abruptly halting LLIN distribution can result in major epidemics with peaks in episode counts and DALYs up to three times the pre-intervention level. This is a result of reduced levels of acquired immunity in the population, while the vector population recovers quickly to the pre-intervention level. Roughly 90% of the population in Africa lives in settings with a transmission below 64 IBPAPA prior to intervention and are most at risk of such large epidemics; epidemics would be less severe in areas with a transmission above 64 IBPAPA prior to intervention. Continuing a high level of CM could somewhat mitigate these epidemics.

The analysis presented here was repeated for children under five years of age only [Additional file 3]. In this analysis, the pre-intervention EIR up to which LLINs are cost-effective is four- to ten-fold higher than in the analysis for the entire population and rebounds after halting distributions are much less severe. This is because in children, acquired immunity is less important than it is for older people, simply because children have been exposed for a shorter period of time and thus have acquired less immunity. Analyses that ignore older age groups [37] could seriously underestimate the implications of waning immunity in the population described here.

Even though more data is becoming available on the temporal dynamics of malaria in specific settings with an increasing history of LLIN distribution [45,46], fitting these malaria models to such temporal data as a means of model validation remains a challenge, with continuously evolving mosquitoes, parasites, therapeutic drug regimens, access to treatment, etc. influencing the temporal dynamics.

This study indicated that scaling up and maintaining high levels of access to effective care can preserve the cost-effectiveness of LLINs in higher transmission settings. Although this simulation study looked only at combinations of CM and LLINs, high levels of access to effective care will be important for mitigating potential rebounds in disease burden occurring after initial successes of transmission reducing interventions. However, in the face of developing drug-resistance and increasing influx of counterfeit drugs, scaling up and maintaining access to effective care may be challenging.

## Endnotes

^a^ It is worth noting that, because of the importation rate of 10 infections per 1000 people per year into the population (included in the scenario in order to simulate importation from neighbouring areas and in order to prevent malaria from becoming extinct for stochastic reasons at low transmission levels), with low CM, it was difficult to simulate transmission settings below 0.0625 IBPAPA. With a lower infection importation rate, lower transmission settings could be simulated [Additional file 11].

^b^ Note that, in contrast to Figure 5, where outcome variables are shown as a ratio of the results in the non-intervention scenarios, Figure 7 shows results in terms of an absolute difference. Whereas in Figures 5, a curve is below the line where ‘ratio equals unity’ indicates a desired effect, in Figure 7, a curve above the horizontal line of ‘zero NHB’ indicates a desired effect. Also, instead of lines for each of the 14 model variants, a goodness of fit-weighted average of the model ensemble is plotted.

^c^ Note that, in contrast to the black line in Figure 7, these plots are for the NHB of LLINs only and the transmission level on the horizontal axis is the one obtained with CM.

^d^ In many African settings, LLIN effectiveness may be different from the effectiveness assumed in this modelling study and the pre-intervention EIR below which LLINs become cost-effective may be different due to differences in mosquito host seeking parameters, seasonality, and other parameters used. Also, even though the 80% reported treatment of recalled fevers is lower than the Roll Back Malaria (RBM) 2010 target, it is still much higher than levels currently being achieved across Africa [47]. Given obstacles to care seeking and ACT access [48,49], such high CM coverage levels might not be realistically achievable in many countries in the near future. Therefore, the exact EIR indicated by these simulation results as the point where LLINs might have negative NHB may not be true in reality.

## Abbreviations

ACT: Artemisinin combination therapy; ANC: Antenatal campaign; BMGF: Bill and Melinda Gates Foundation; BOINC: Berkeley open infrastructure for network computing; CM: Case management; DALYs: Disability adjusted life years; DHS: Demographic and health survey; EIR: Entomological inoculation rate; EPI: Expanded programme on immunization; IBPAPA: Infectious bites per adult per annum; ICERs: Incremental cost-effectiveness ratios; ITN: Insecticide-treated nets; MIS: Malaria indicator survey; NHB: Net health benefits; RBM: Roll back malaria; SP: Sulphadoxine-pyrimethamine; USAID: United States agency for international development; WHO: World health organization.

## Competing interests

The authors declare that they have no competing interests.

## Authors’ contributions

OJTB designed the study, ran the simulations, analysed the results and drafted the manuscript. MAP provided expert advice on model fitting and helped draft the manuscript. Both authors approved the final manuscript.

## Supplementary Material

Additional file 1 ICERs of LLINs and scaled-up CM. Click here for file

Additional file 2 Central scenario. Click here for file

Additional file 3 Figures for children under five years of age. Click here for file

Additional file 4 Infectiousness of the human population to mosquitoes. Click here for file

Additional file 5 NHB cumulative over time. Click here for file

Additional file 6 Summary over the period 13 – 16 years since the start of the simulation. Click here for file

Additional file 7 Sensitivity of model fit to data presented by Marsh and Snow. Click here for file

Additional file 8 Sensitivity analysis. Click here for file

Additional file 9 Impact of LLIN distributions on EIR. Click here for file

Additional file 10 Impact of halting LLIN distribution in scenarios with high CM coverage relative to continued high CM coverage. Click here for file

Additional file 11 Effect of imported infections. Click here for file
