# Supplementary material for: Repeated mass distributions and continuous distribution of long-lasting insecticidal nets: modelling sustainability of health benefits from mosquito nets, depending on case management
Source: Malar J. 2013 Nov 7;12:401. doi: 10.1186/1475-2875-12-401 (PMC4228503; doi:10.1186/1475-2875-12-401)
Supplement: Additional file 1 — ICERs of LLINs and scaled-up CM. [file 1475-2875-12-401-S1.pdf]

Additional file 1: ICERs of LLINs and scaled-up CM.

White and colleagues [7] provide a literature review of cost effectiveness of LLINs, indoor residual spraying and artemisinin combination therapy (ACT), and find provider ICER interquartile ranges of 15 – 46 and 13 – 37 United States $[2009] per DALY for LLINs and ACT, respectively. Figure S1.1 provides model based estimates of provider ICER of LLIN and scaled-up case management during the first four years of implementation. ICERs were highly dependent on the pre-intervention EIR, and, as a result, varied over a much wider range than observed by White and colleagues. At a pre-intervention EIR of four IBPAPA, the ICER for LLINs as a single intervention was predicted to be negative. This is because the cost of curative treatment prevented by LLINs exceeds the distribution cost of LLINs in that situation. In higher transmission settings (roughly, pre-intervention EIRs above 32 IBPAPA), DALYs averted are small, resulting in larger ICER ranges. The ICER for scaled-up CM increases with increasing pre-intervention EIR and levels off at levels higher than 16 IBPAPA. Above 16 IBPAPA, CM reduces mainly the disease burden associated with (new) infections, and the impact on transmission itself is negligible. At lower transmission settings, scaled-up CM also reduces transmission and ensuing episodes (see also Figure 2 and Figure 5 in the main manuscript).

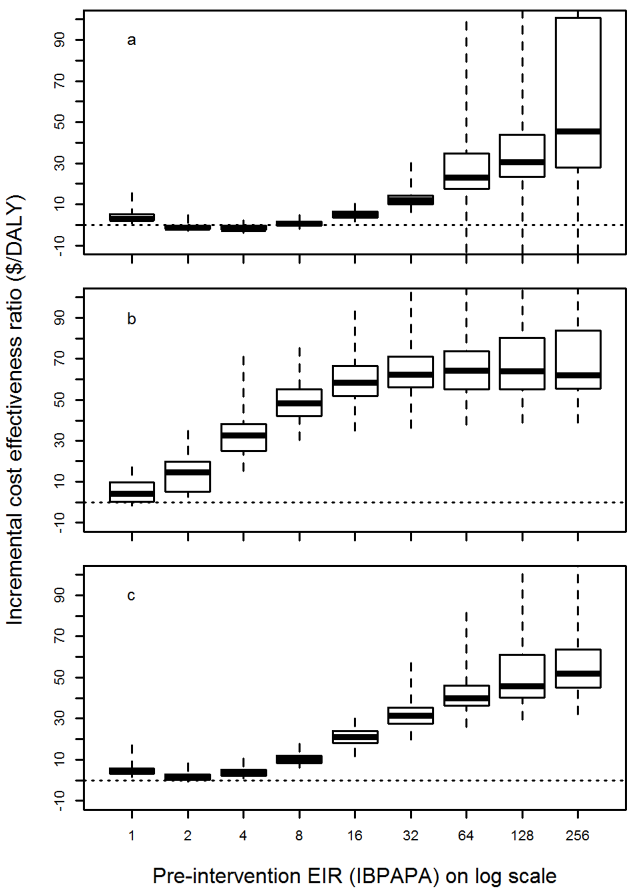

Figure S1.1 Box plot of incremental cost effectiveness ratio of LLINs and CM. Incremental cost effectiveness ratios are for provider cost in 2012 United States $ per disability adjusted life year (DALY) averted depending on the pre-intervention entomological rate expressed in infectious bites per adult per annum (IBPAPA) during the first four years of implementation of a) LLINs distributed to 63% of the human population in mass distributions, augmented with LLINs distributed to 72% of new-borns through neo-natal services, b) scaling up case management from 9% reported treatment of recalled fevers with an effective antimalarial drug in Demographic and Health Surveys to 80%, and c) LLINs and scaled up case management. Boxes depict interquartile range, horizontal bars the median, and vertical dashed lines depict the range of results of simulations using 14 model variants with 10 stochastic seeds each.
