# Supplementary material for: Repeated mass distributions and continuous distribution of long-lasting insecticidal nets: modelling sustainability of health benefits from mosquito nets, depending on case management
Source: Malar J. 2013 Nov 7;12:401. doi: 10.1186/1475-2875-12-401 (PMC4228503; doi:10.1186/1475-2875-12-401)
Supplement: Additional file 3 — Figures for children under five years of age. [file 1475-2875-12-401-S3.pdf]

Additional file 3: Figures for children under five years of age.

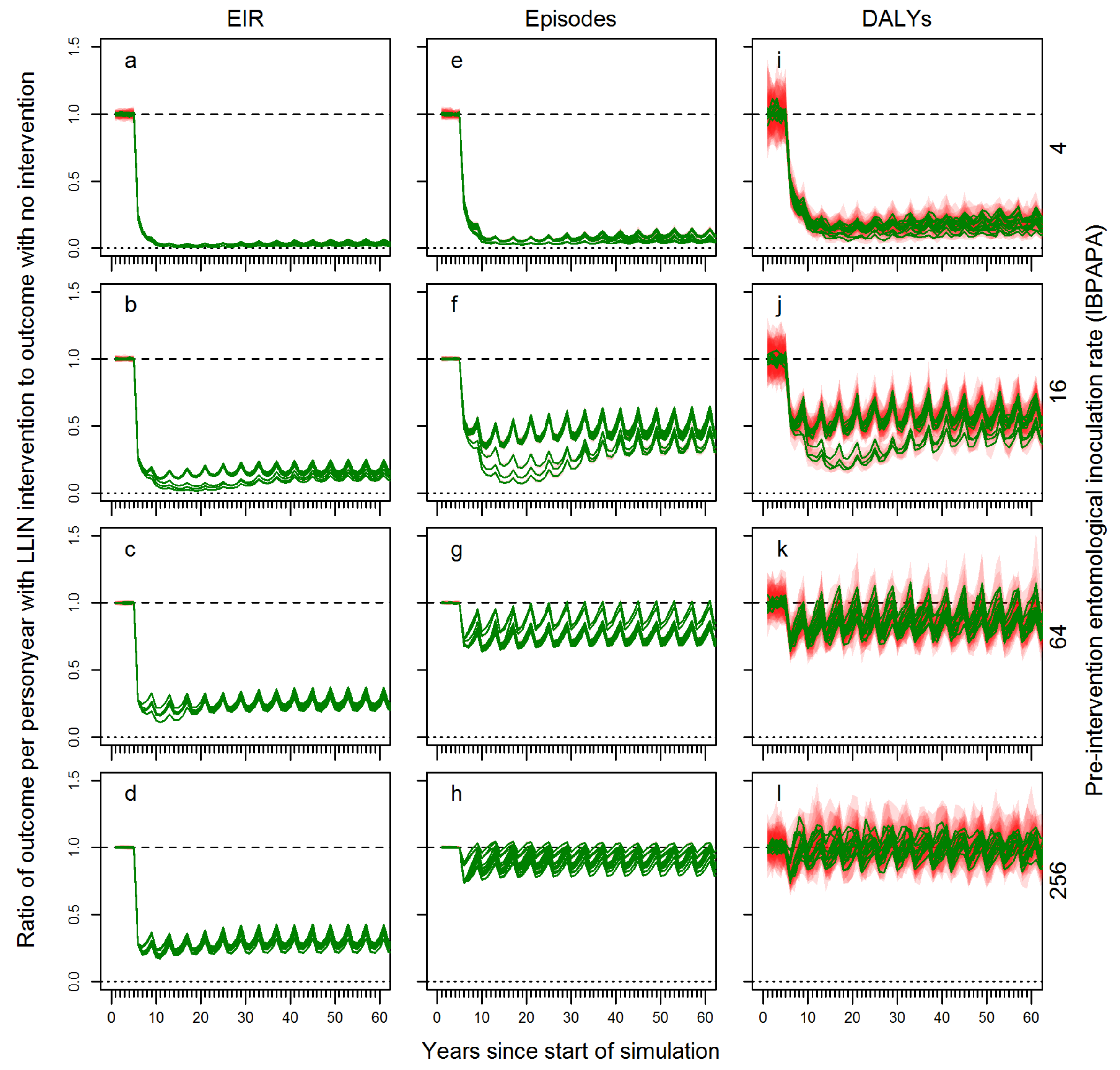

Figure S3.1 Impact of LLINs on EIR, and episodes and DALYs for children under five years of age, over time. Green lines show medians and red polygons show ranges over 10 runs with unique seeds, of 14 model variants, of the ratio of the outcome variable in the intervention scenario relative to in the reference scenario (without intervention). **a – d**) entomological inoculation rate (EIR), **e – g**) episodes and **i – l**) disability adjusted life years (DALYs). Rows of panels have the same pre-intervention EIR.

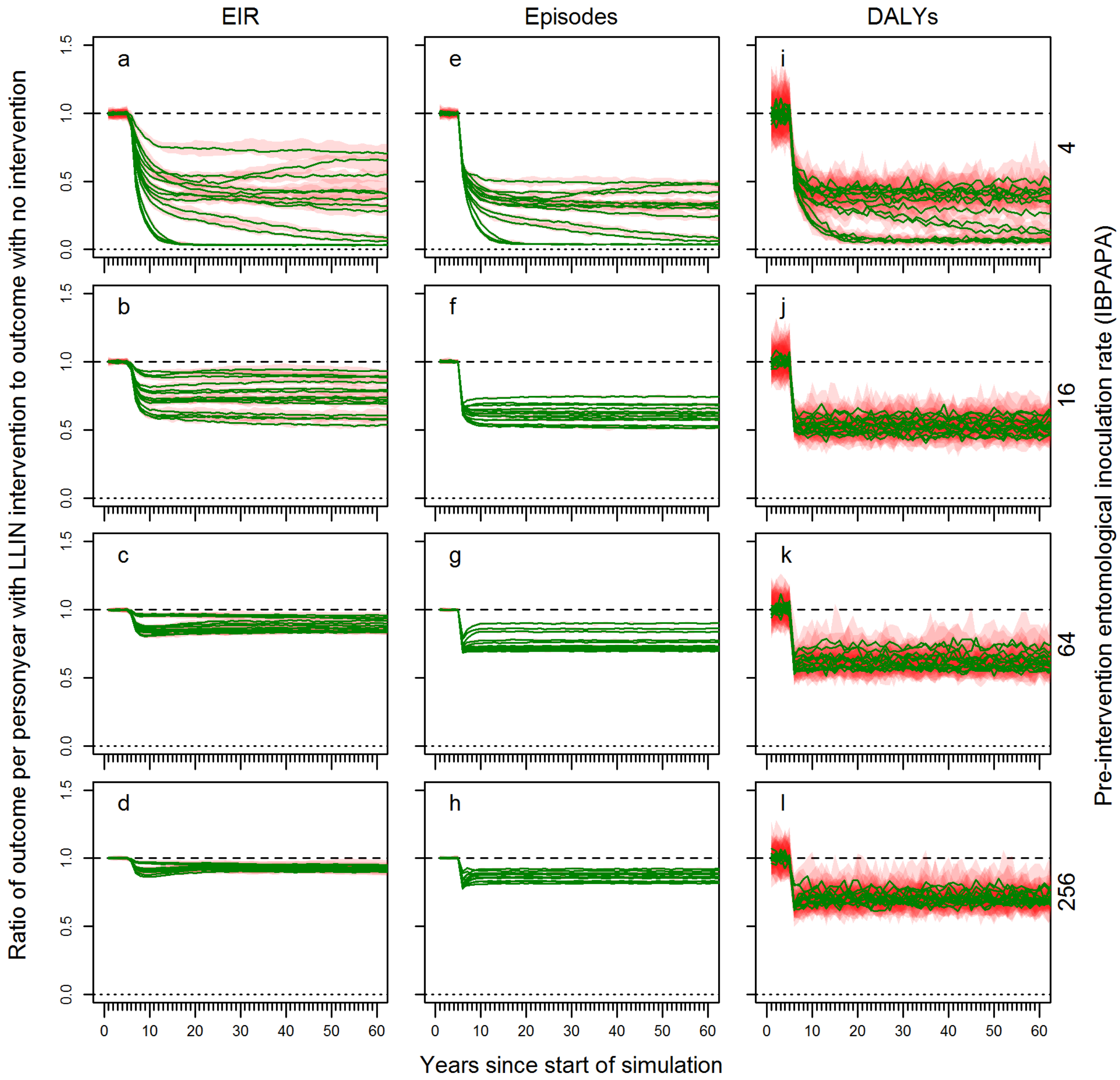

Figure S3.2 Impact of scaled-up case management on EIR, and episodes and DALYs for children under five years of age, over time. See legend of Figure S3.1.

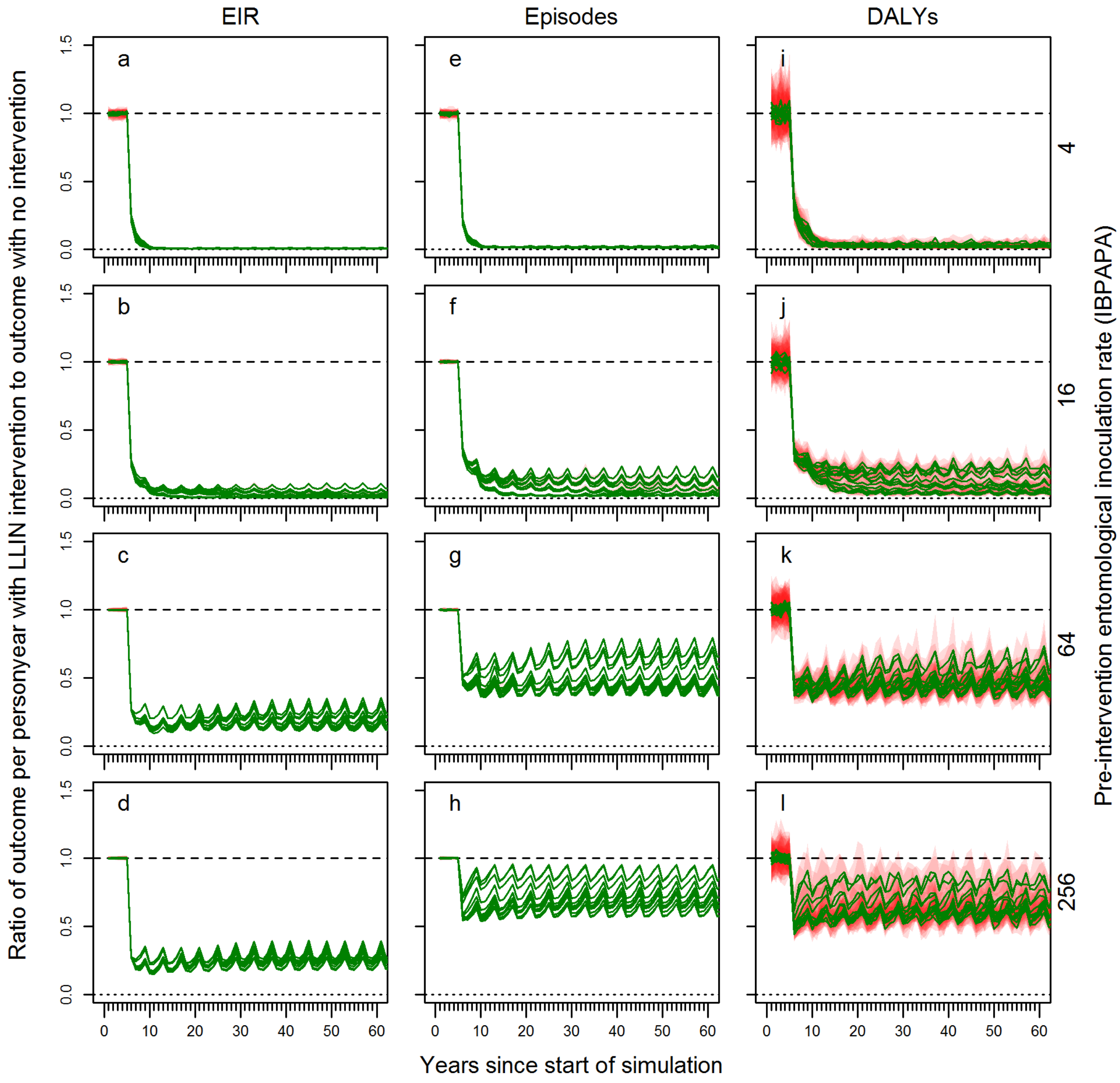

Figure S3.3 Impact of LLIN distributions and scaled-up case management on EIR, and episodes and DALYs for children under five years of age, over time. See legend of Figure S3.1.

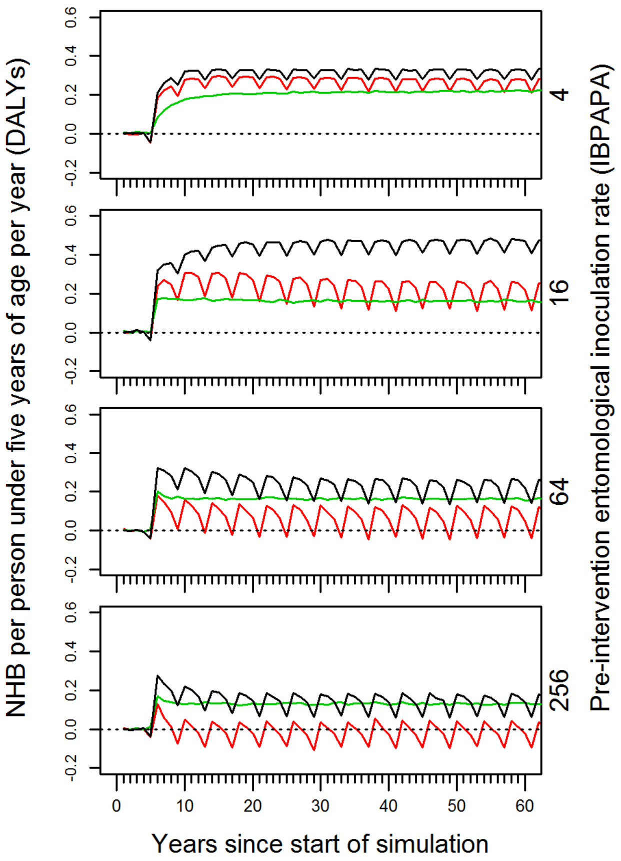

Figure S3.4 Temporal dynamics of net health benefits of LLINs and case management for children under five years of age. Lines are goodness of fit-weighted averages of the 14 variants in the model ensemble. Net health benefits are calculated as compared to scenarios with a low baseline case management (CM) of 9% reported treatment of recalled fevers with an effective antimalarial drug in demographic health surveys or similar surveys. Red lines show the effect of only distributing long lasting insecticidal nets (LLINs). Green lines show the effect of only scaling up CM to 80% reported treatment of recalled fevers. Black lines show the effect of both distributing LLINs and scaling up CM to 80%.

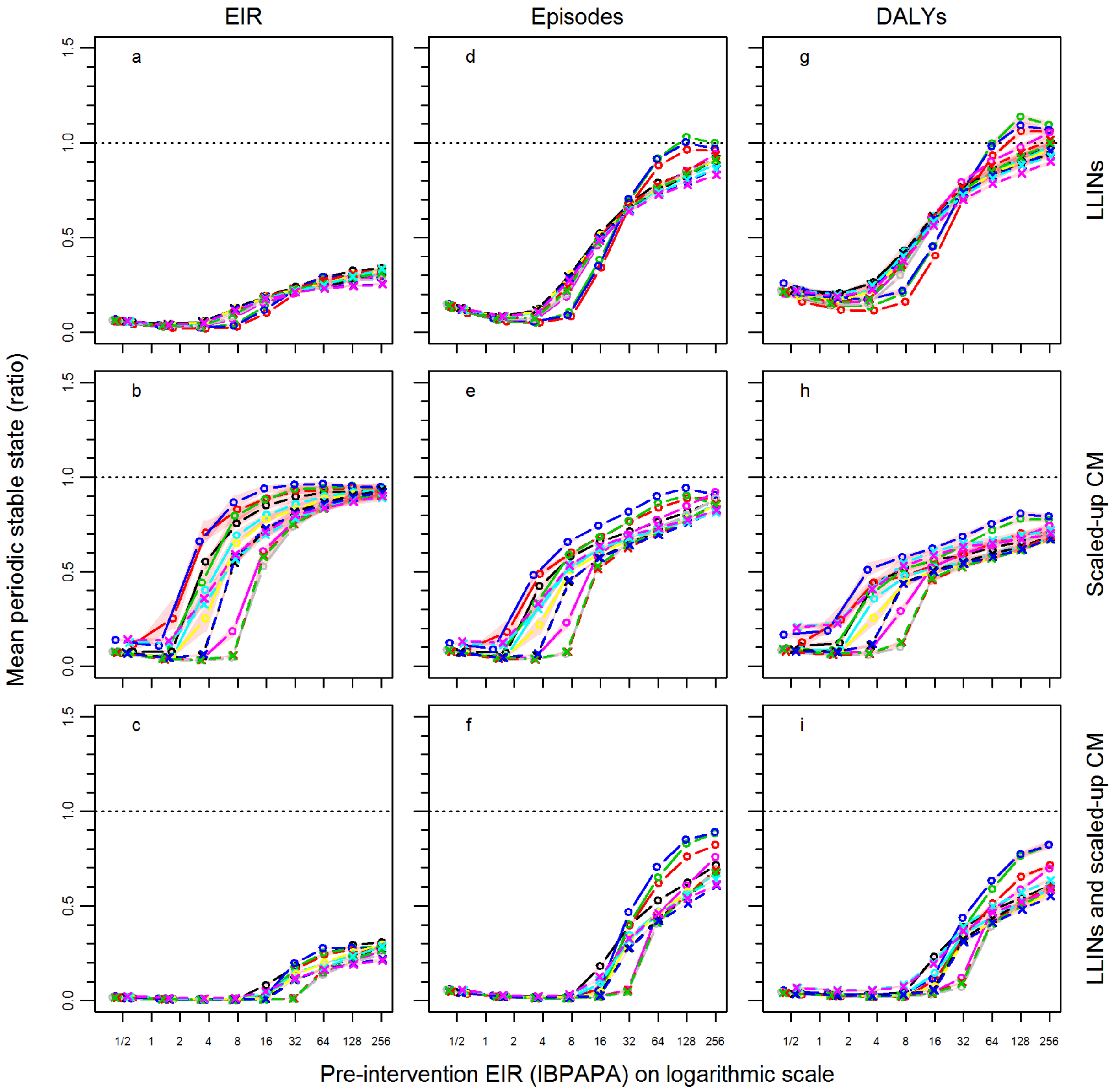

Figure S3.5 Impact of LLINs, CM or both on EIR, and episodes and DALYs for children under five years of age, at periodic stable state. Ratios of results for intervention scenarios (**a**, **d** and **g**: long lasting insecticidal nets (LLINs); **b**, **e** and **h** scaled up case management (CM); and **c**, **f** and **i** both LLINs and scaled-up CM) and non-intervention scenarios (low CM only) calculated for means over the last 60 years of individual runs of 125 years, with 10 unique seeds per input EIR and model variant combination, for outcomes **a**, **b** and **c:** entomological inoculation rate (EIR): **d**, **e** and **f**: episodes; and **g**, **h** and **i**: disability adjusted life years (DALYs). Lines connect median values of groups of the ten seeds with the same input EIR and model variant. Model variants [17]: R0001 = solid black lines and circles; R0063 = solid red lines and circles; R0065 = solid lime green lines and circles; R0068 = solid blue lines and circles; R0111 = solid cyan lines and circles; R0115 = solid magenta lines and circles; R0121 = solid yellow lines and circles; R0125 = solid grey lines and circles; R0131 = dashed black lines and crosses; R0132 = dashed red lines and crosses; R0133 = dashed lime green lines and crosses; R0670 = dashed blue lines and crosses; R0674 = dashed cyan lines and crosses; R0678 = dashed magenta lines and crosses. Red polygons show ranges.

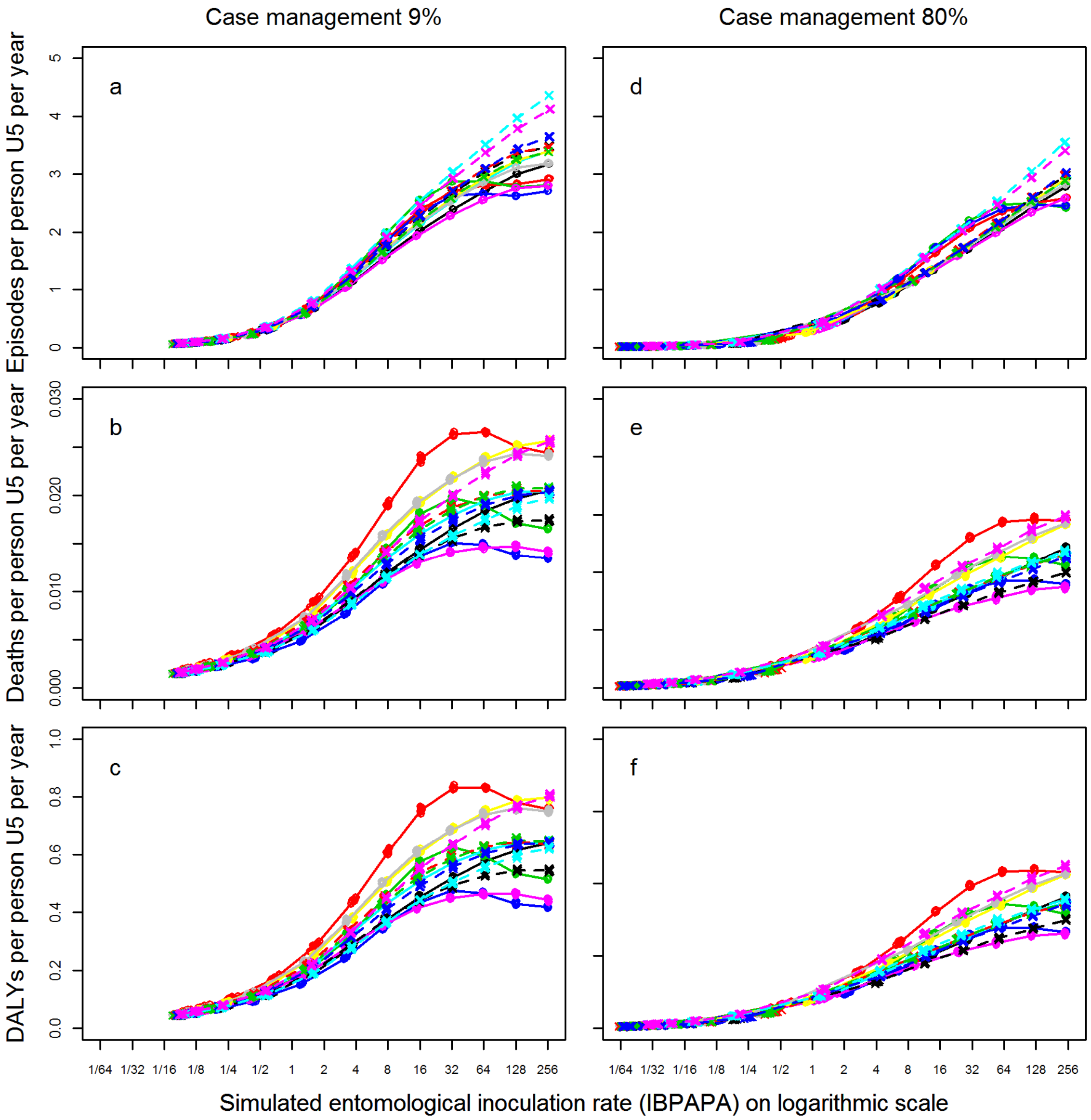

Figure S3.6 Episodes, deaths, and DALYs depending on transmission and CM without LLINs in the children under five years of age. Averages for the last 60 years of individual runs of 125 years, with 10 unique seeds per input EIR and model variant combination, with **a & d**) episodes per person per year **b & e**) direct and indirect deaths due to malaria, and **c & f**) disability adjusted life years (DALYs). Lines connect median values of groups of the ten seeds with the same input EIR and model variant. See the legend of Figure S3.5 for the colour coding of model variants [17].

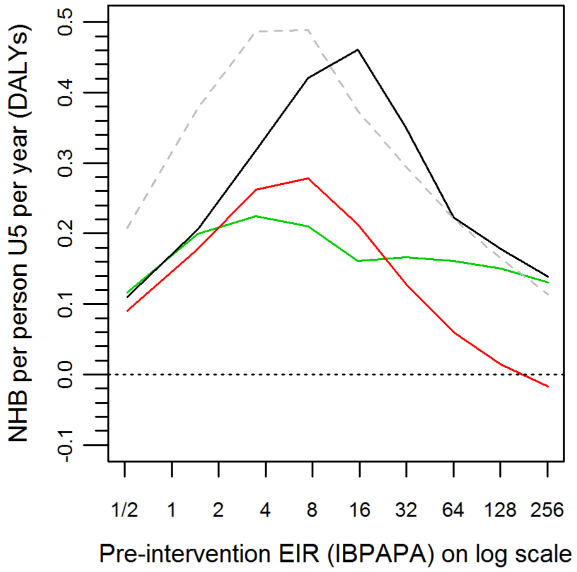

Figure S3.7 Mean NHBs of LLINs and CM for children under five years of age at periodic stable state. Lines are goodness of fit-weighted averages of the 14-variant model ensemble, averaged over the last 60 years of the 125 year simulation period, where the net health benefits (NHB) are in a periodic stable state. NHB are calculated as compared to scenarios with a low baseline case management (CM) of 9% reported treatment of recalled fevers with an effective antimalarial drug in DHS type surveys. The red line shows the effect of only distributing LLINs. The green line shows the effect of only scaling up CM to 80% reported treatment of recalled fevers. The black line shows the effect of both distributing LLINs and scaling up CM to 80%. The grey dashed line is the sum of the red and green line.

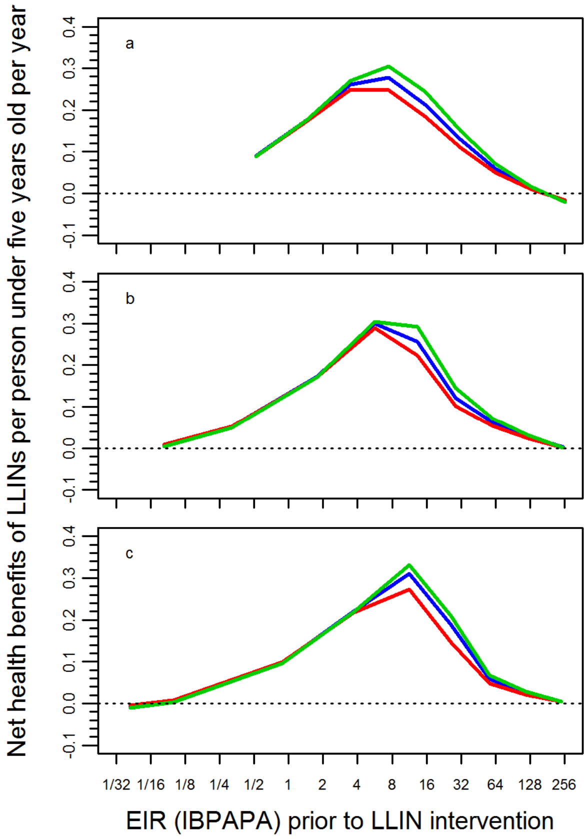

Figure S3.8 NHBs of LLINs for children under five years of age depending on CM and distribution rate. Lines are goodness of fit-weighted averages of the 14 variant model ensemble, averaged over the last 60 years of the 125 year simulations. Green, blue and red lines represent scenarios where mass distributions were done every three, four and five years, respectively. **a**) Case management (CM) level of 9% reported treatment of recalled fevers with an effective antimalarial drug DHS type surveys. **b**) CM level of 59%. **c**) CM level of 80%.

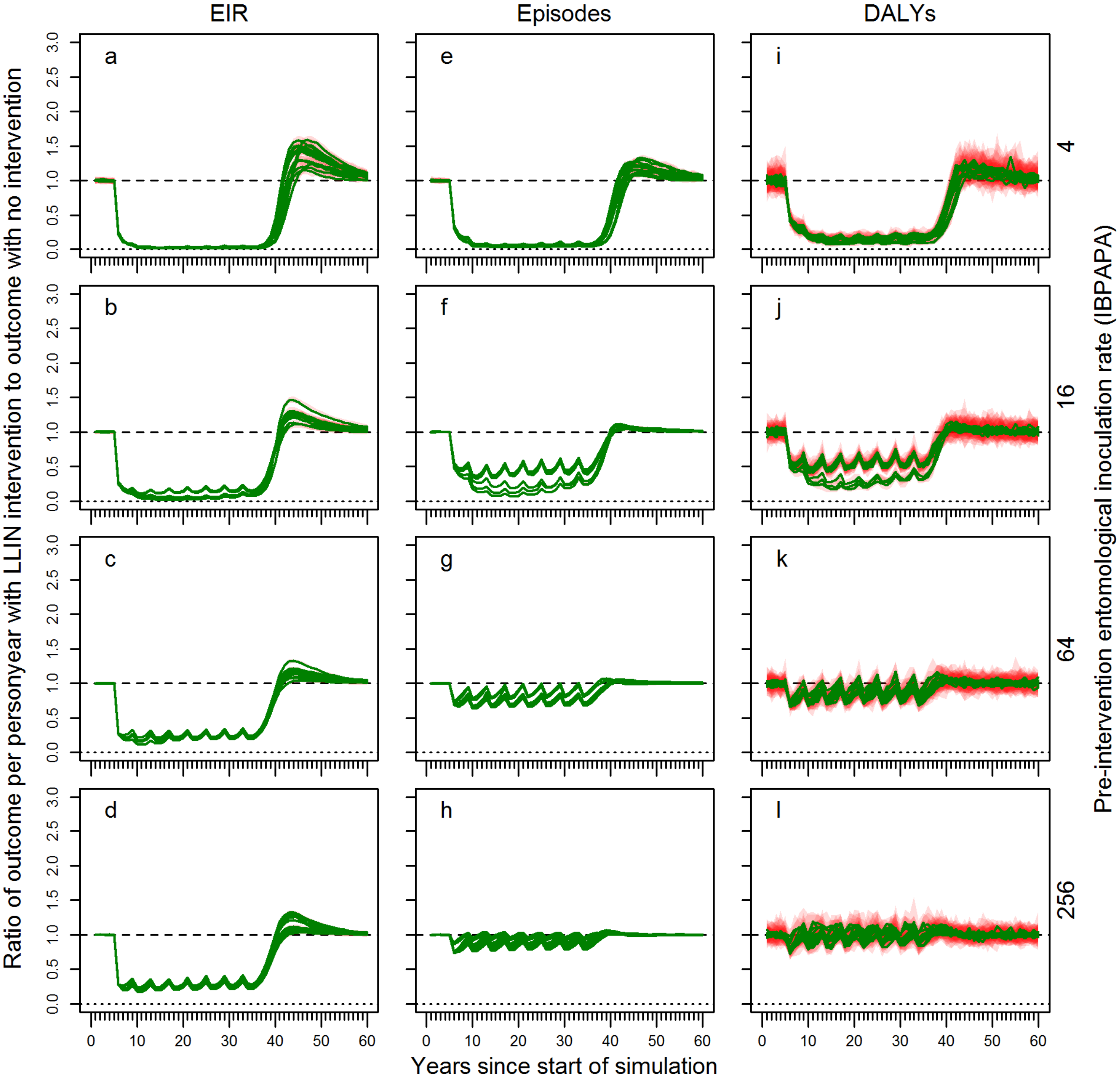

Figure S3.9 Impact of halting LLIN distribution on EIR, and episodes and DALYs in children under five years of age for a central scenario over time. See legend of Figure S3.1.

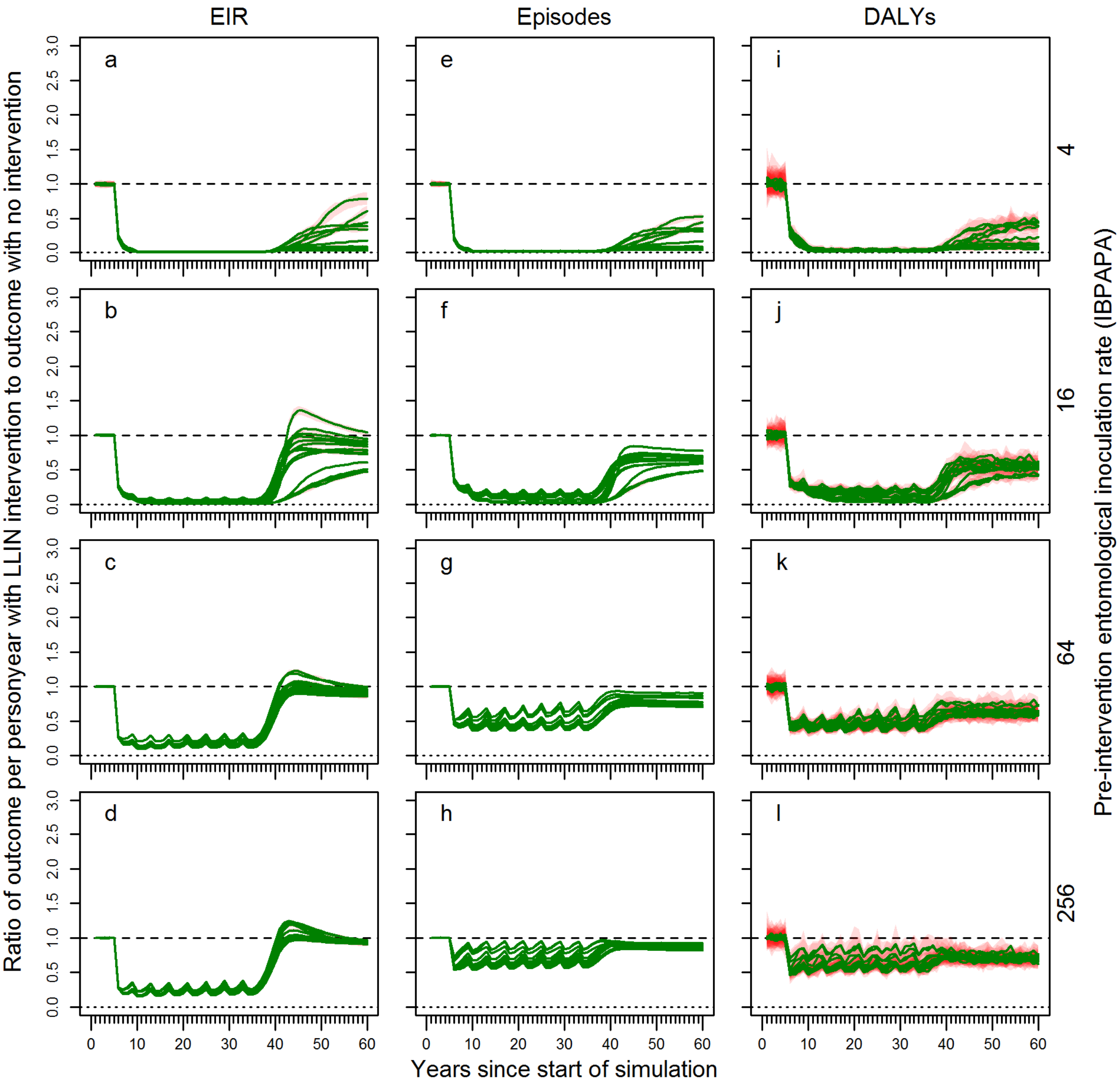

Figure S3.10 Impact of halting LLIN distribution on EIR, and episodes and DALYs in children under five years of age, with continued scaled-up CM. See legend of Figure S3.1.

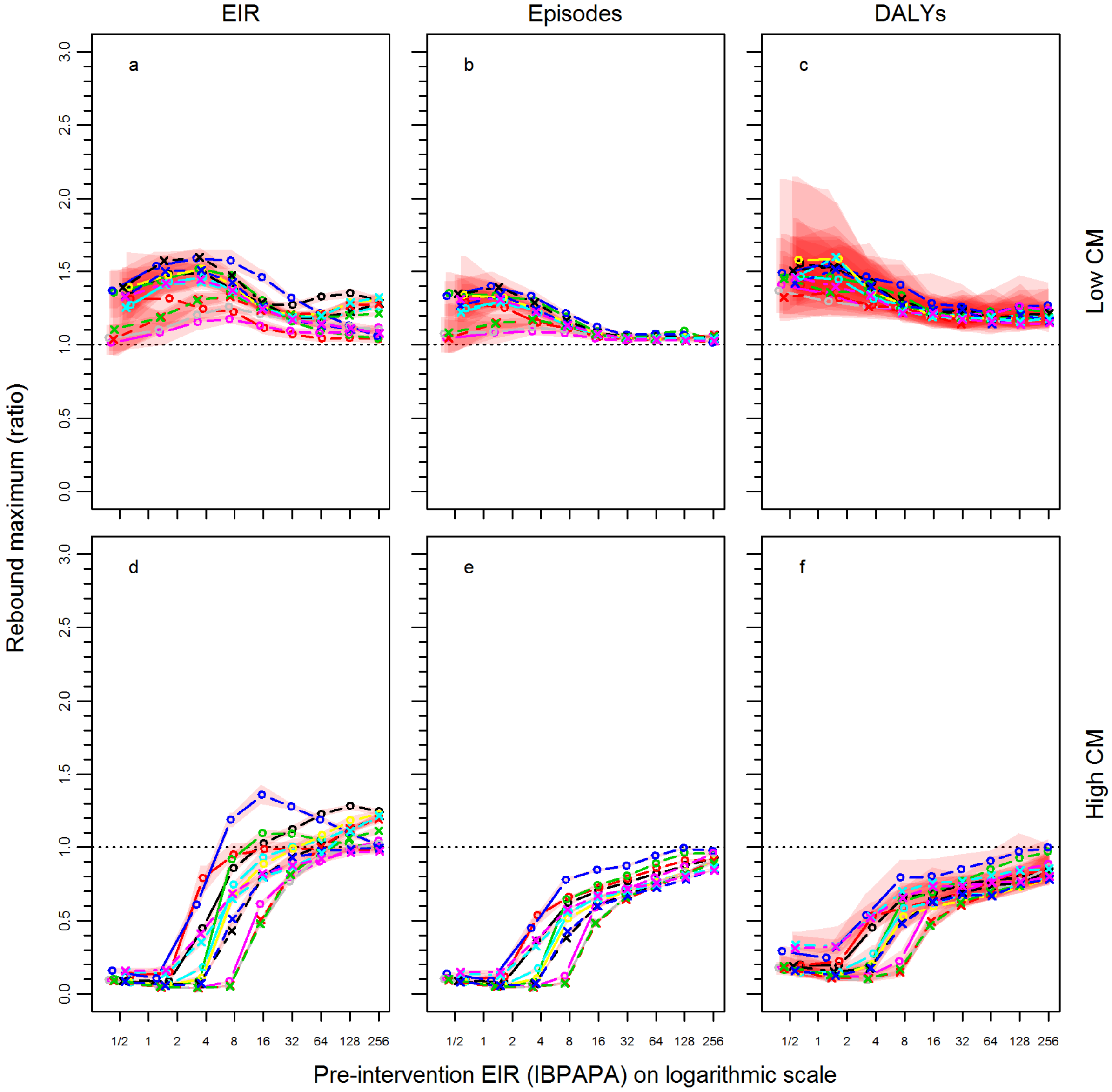

Figure S3.11 Maximum rebound in EIR, and in episodes and DALYs in children under five years of age after abruptly halting LLIN distributions, depending on CM and pre-intervention EIR. Maxima in the ratios of results after halting LLIN distribution after eight rounds (with **a**, **b** and **c**: low baseline CM and **d**, **e** and **f** scaled-up CM); and non-intervention scenarios (low CM only) calculated for means over the last 60 years of individual runs of 125 years, with 10 unique seeds per input EIR and model variant combination, for outcomes **a** and **d:** entomological inoculation rate (EIR): **b**, and **e**: episodes; and **c** and **f**: disability adjusted life years (DALYs). Lines connect median values of groups of the ten seeds with the same input EIR and model variant. See the legend of Figure S3.5 for model variant [17] colour coding. Red polygons show ranges. Note that if the rebound maximum is shown is below 1.0, it is possible that the maximum is not reached within 23 years post halting of LLIN distribution, and it is likely that in that case, the maximum is not larger than 1.0.
