# Supplementary material for: Repeated mass distributions and continuous distribution of long-lasting insecticidal nets: modelling sustainability of health benefits from mosquito nets, depending on case management
Source: Malar J. 2013 Nov 7;12:401. doi: 10.1186/1475-2875-12-401 (PMC4228503; doi:10.1186/1475-2875-12-401)
Supplement: Additional file 4 — Infectiousness of the human population to mosquitoes. [file 1475-2875-12-401-S4.pdf]

Additional file 4: Infectiousness of the human population to mosquitoes.

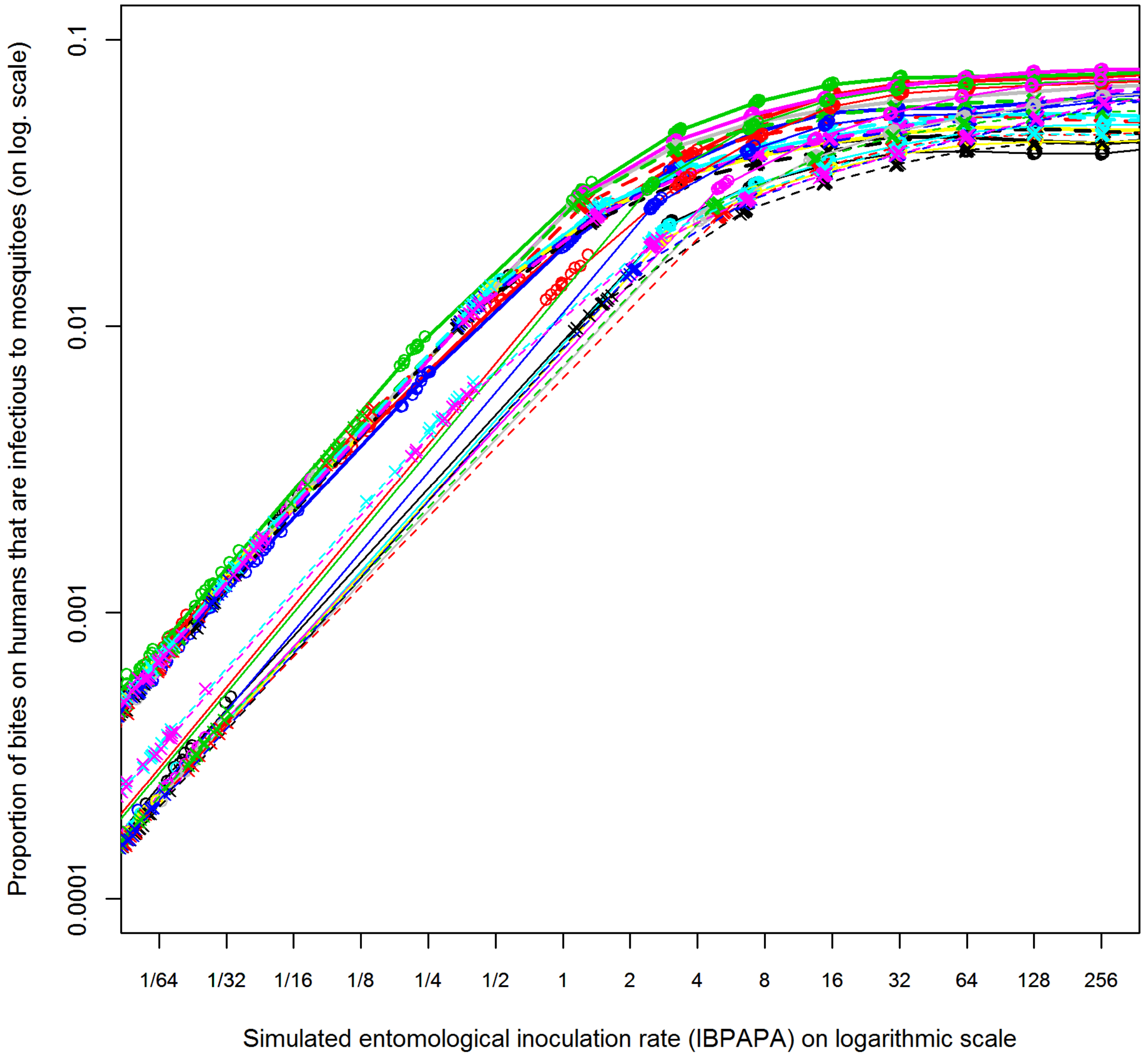

Figure S4.1 Proportion of bites on humans that are infectious to mosquitoes depending on transmission and CM (without LLINs).

Lines connect median values of groups of the 10 seeds with the same input EIR and model variant. Thick lines are for 9% CM coverage and thick lines are for 80% CM coverage. Model variants [17]: R0001 = solid black lines and circles; R0063 = solid red lines and circles; R0065 = solid lime green lines and circles; R0068 = solid blue lines and circles; R0111 = solid cyan lines and circles; R0115 = solid magenta lines and circles; R0121 = solid yellow lines and circles; R0125 = solid grey lines and circles; R0131 = dashed black lines and crosses; R0132 = dashed red lines and crosses; R0133 = dashed lime green lines and crosses; R0670 = dashed blue lines and crosses; R0674 = dashed cyan lines and crosses; R0678 = dashed magenta lines and crosses.
