# Supplementary material for: Repeated mass distributions and continuous distribution of long-lasting insecticidal nets: modelling sustainability of health benefits from mosquito nets, depending on case management
Source: Malar J. 2013 Nov 7;12:401. doi: 10.1186/1475-2875-12-401 (PMC4228503; doi:10.1186/1475-2875-12-401)
Supplement: Additional file 5 — NHB cumulative over time. [file 1475-2875-12-401-S5.pdf]

Additional file 5: NHB cumulative over time

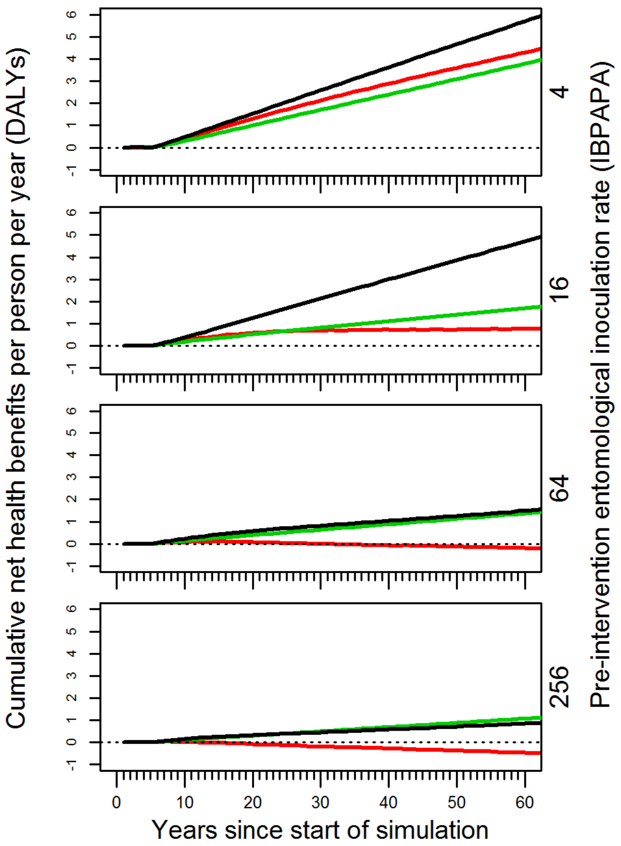

Figure S5.1 Temporal dynamics of net health benefits of LLINs and case management, cumulated of time. Lines are goodness of fit-weighted averages of the 14 variants in the model ensemble. Net health benefits are calculated as compared to scenarios with a low baseline case management (CM) of 9% reported treatment of recalled fevers with an effective antimalarial drug in demographic health surveys or similar surveys. Red lines show the effect of only distributing long lasting insecticidal nets (LLINs). Green lines show the effect of only scaling up CM to 80% reported treatment of recalled fevers. Black lines show the effect of both distributing LLINs and scaling up CM to 80%.

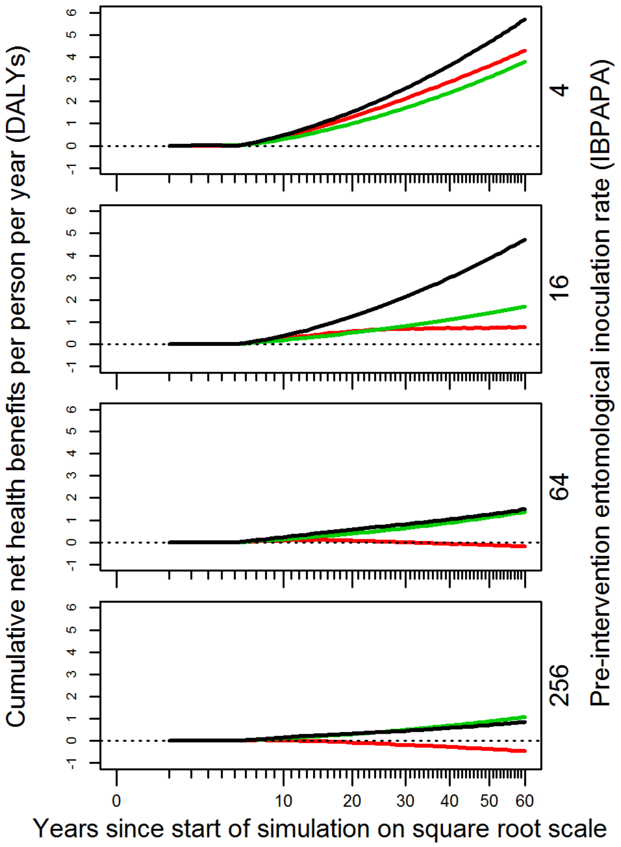

Figure S5.2 Temporal dynamics of net health benefits of LLINs and case management, cumulated of time, with time on square root scale. The time on the horizontal axis is plotted on a square root scale. See further the legend of Figure S5.1.

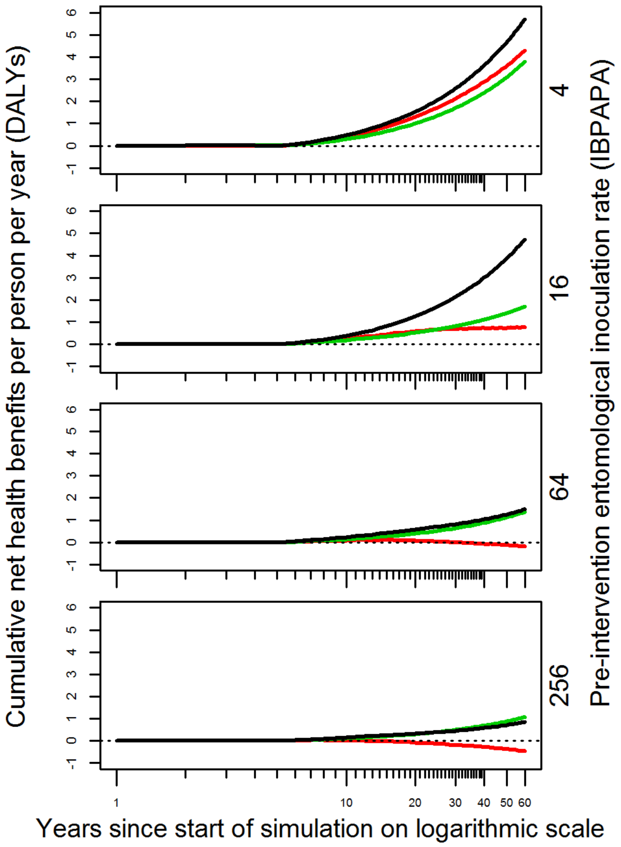

Figure S5.3 Temporal dynamics of net health benefits of LLINs and case management, cumulated of time, with time on logarithmic root scale. The time on the horizontal axis is plotted on a logarithmic scale. See further the legend of Figure S5.1.
