# Supplementary material for: Repeated mass distributions and continuous distribution of long-lasting insecticidal nets: modelling sustainability of health benefits from mosquito nets, depending on case management
Source: Malar J. 2013 Nov 7;12:401. doi: 10.1186/1475-2875-12-401 (PMC4228503; doi:10.1186/1475-2875-12-401)
Supplement: Additional file 6 — Summary over the period 13 – 16 years since the start of the simulation. [file 1475-2875-12-401-S6.pdf]

Additional file 6: Summary over the period 13 – 16 years since the start of the simulation

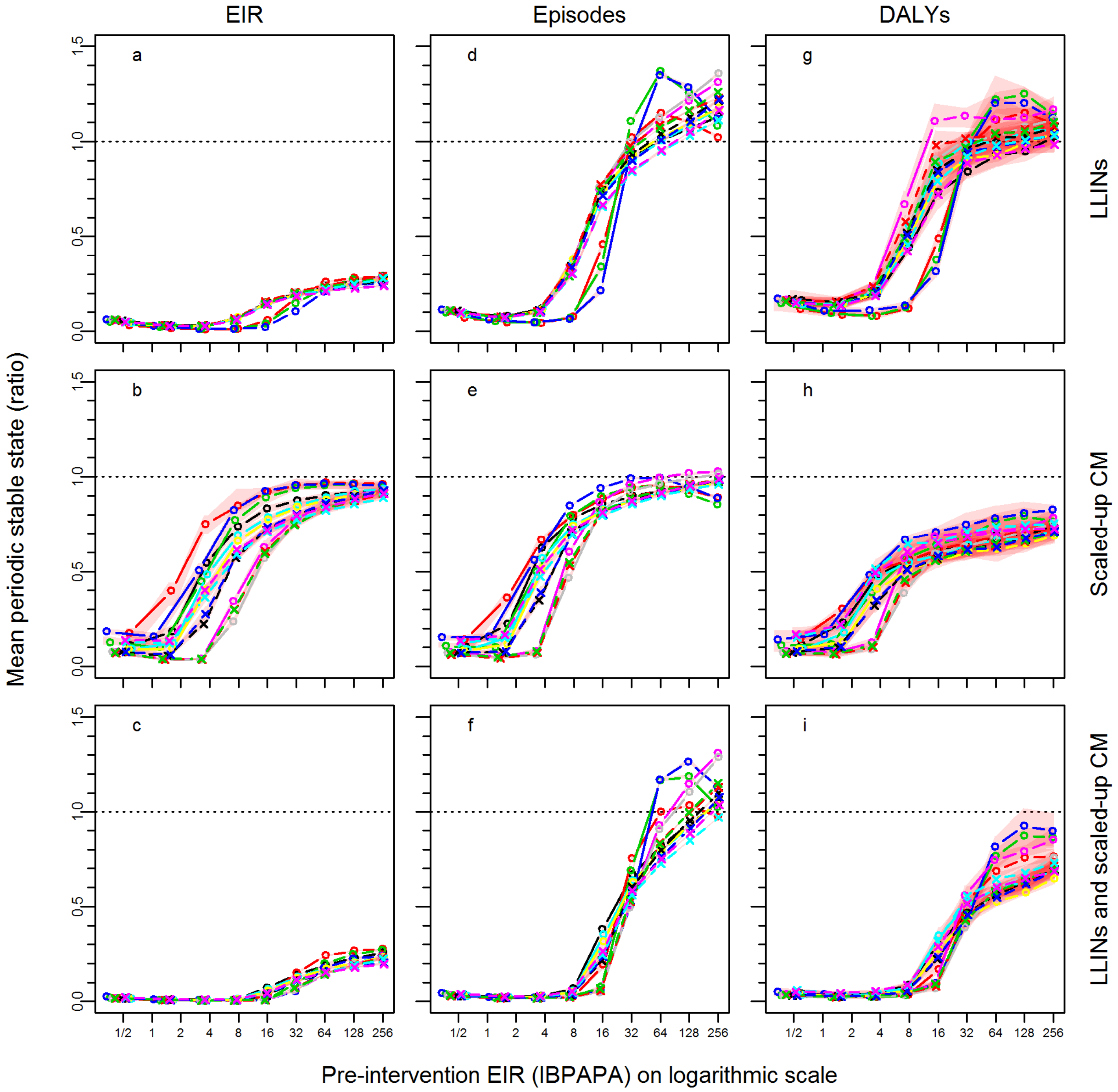

Figure S6.1 Impact of LLINs, CM or both on EIR, episodes and DALYs during the fourth distribution round. Ratios of results for intervention scenarios (**a**, **d** and **g**: long lasting insecticidal nets (LLINs); **b**, **e** and **h** scaled up case management (CM); and **c**, **f** and **i** both LLINs and scaled-up CM) and non-intervention scenarios (low CM only) calculated for means over the last 60 years of individual runs of 125 years, with 10 unique seeds per input EIR and model variant combination, for outcomes **a**, **b** and **c:** entomological inoculation rate (EIR): **d**, **e** and **f**: episodes; and **g**, **h** and **i**: disability adjusted life years (DALYs). Lines connect median values of groups of the ten seeds with the same input EIR and model variant. Model variants [17]: R0001 = solid black lines and circles; R0063 = solid red lines and circles; R0065 = solid lime green lines and circles; R0068 = solid blue lines and circles; R0111 = solid cyan lines and circles; R0115 = solid magenta lines and circles; R0121 = solid yellow lines and circles; R0125 = solid grey lines and circles; R0131 = dashed black lines and crosses; R0132 = dashed red lines and crosses; R0133 = dashed lime green lines and crosses; R0670 = dashed blue lines and crosses; R0674 = dashed cyan lines and crosses; R0678 = dashed magenta lines and crosses. Red polygons show ranges.

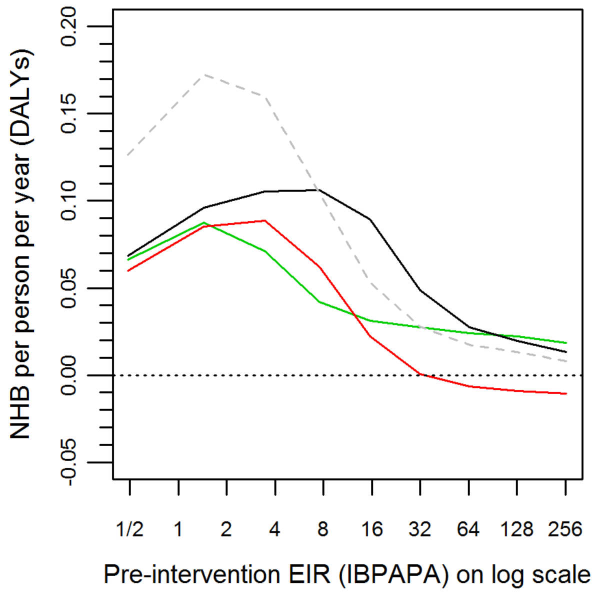

Figure S6.2 Mean NHB of LLINs and CM during the fourth distribution round. Lines are goodness of fit-weighted averages of the 14-variant model ensemble, averaged over the last 60 years of the 125 year simulation period, where the net health benefits (NHB) are in a periodic stable state. NHB are calculated as compared to scenarios with a low baseline case management (CM) of 9% reported treatment of recalled fevers with an effective antimalarial drug in DHS type surveys. The red line shows the effect of only distributing LLINs. The green line shows the effect of only scaling up CM to 80% reported treatment of recalled fevers. The black line shows the effect of both distributing LLINs and scaling up CM to 80%. The grey dashed line is the sum of the red and green line.
