# Supplementary material for: Repeated mass distributions and continuous distribution of long-lasting insecticidal nets: modelling sustainability of health benefits from mosquito nets, depending on case management
Source: Malar J. 2013 Nov 7;12:401. doi: 10.1186/1475-2875-12-401 (PMC4228503; doi:10.1186/1475-2875-12-401)
Supplement: Additional file 7 — Sensitivity of model fit to data presented by Marsh and Snow. [file 1475-2875-12-401-S7.pdf]

Additional file 7: Sensitivity of model fit to data presented by Marsh and Snow

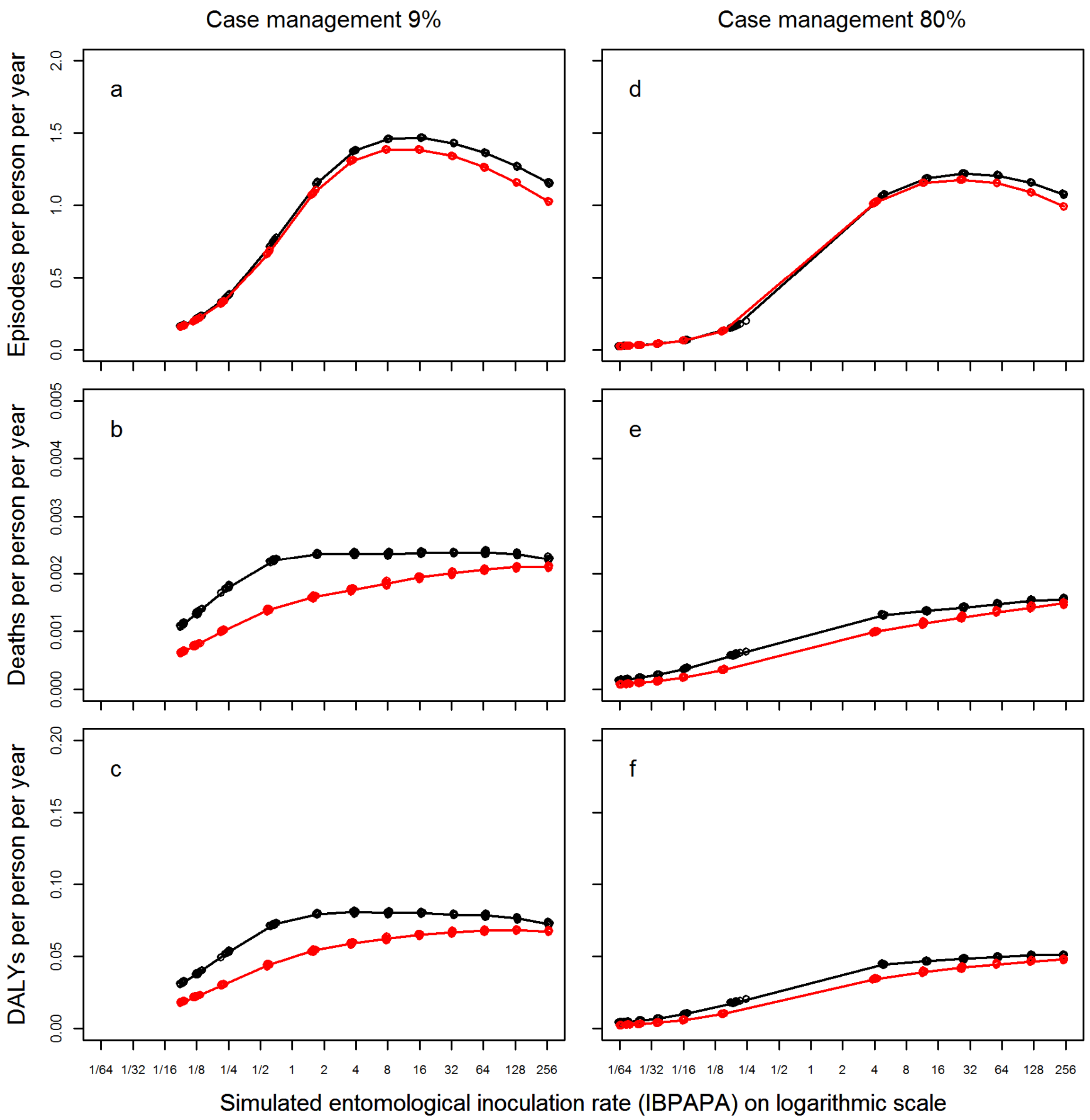

Figure S7.1 Episodes, deaths, and DALYs depending on transmission and CM without LLINs. Averages for the last 60 years of individual runs of 125 years, with 10 unique seeds per input EIR and model variant combination, with **a** & **d**) episodes per person per year **b** & **e**) direct and indirect deaths due to malaria, and **c** & **f**) disability adjusted life years (DALYs). Lines connect median values of groups of the ten seeds with the same input EIR and model variant. Black lines and dots represent model variant R1134 with the data of Marsh and Snow (1999) included in the fitting objectives. Red lines and dots represent model variant R3300, which is has exactly the same specifications as variant R1134 except that the data of Marsh and Snow (1999) were excluded from the fitting objectives.

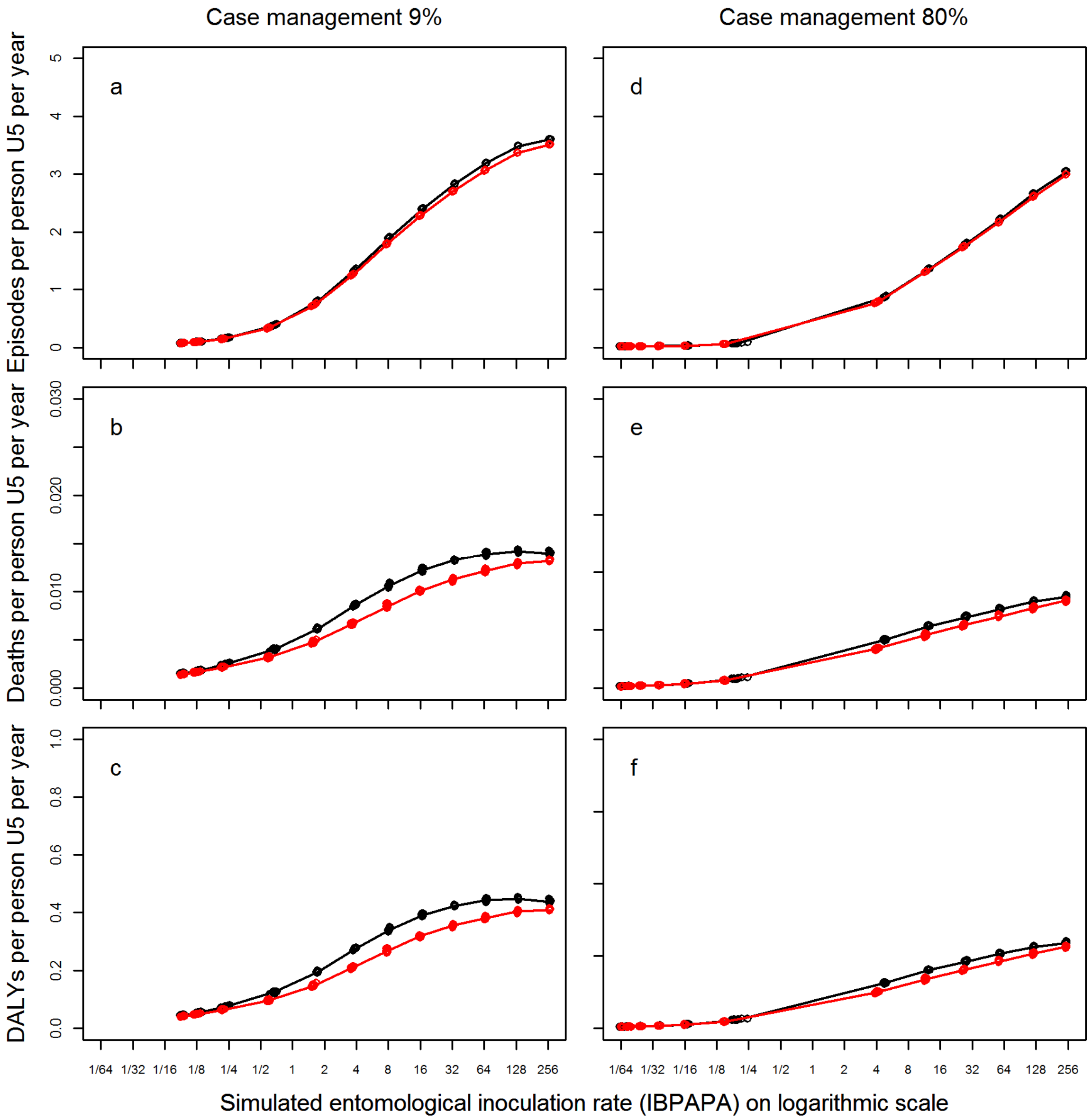

Figure S7.2 Episodes, deaths, and DALYs in for children under five years of age only depending on transmission and CM without LLINs. See legend of Figure S7.1.

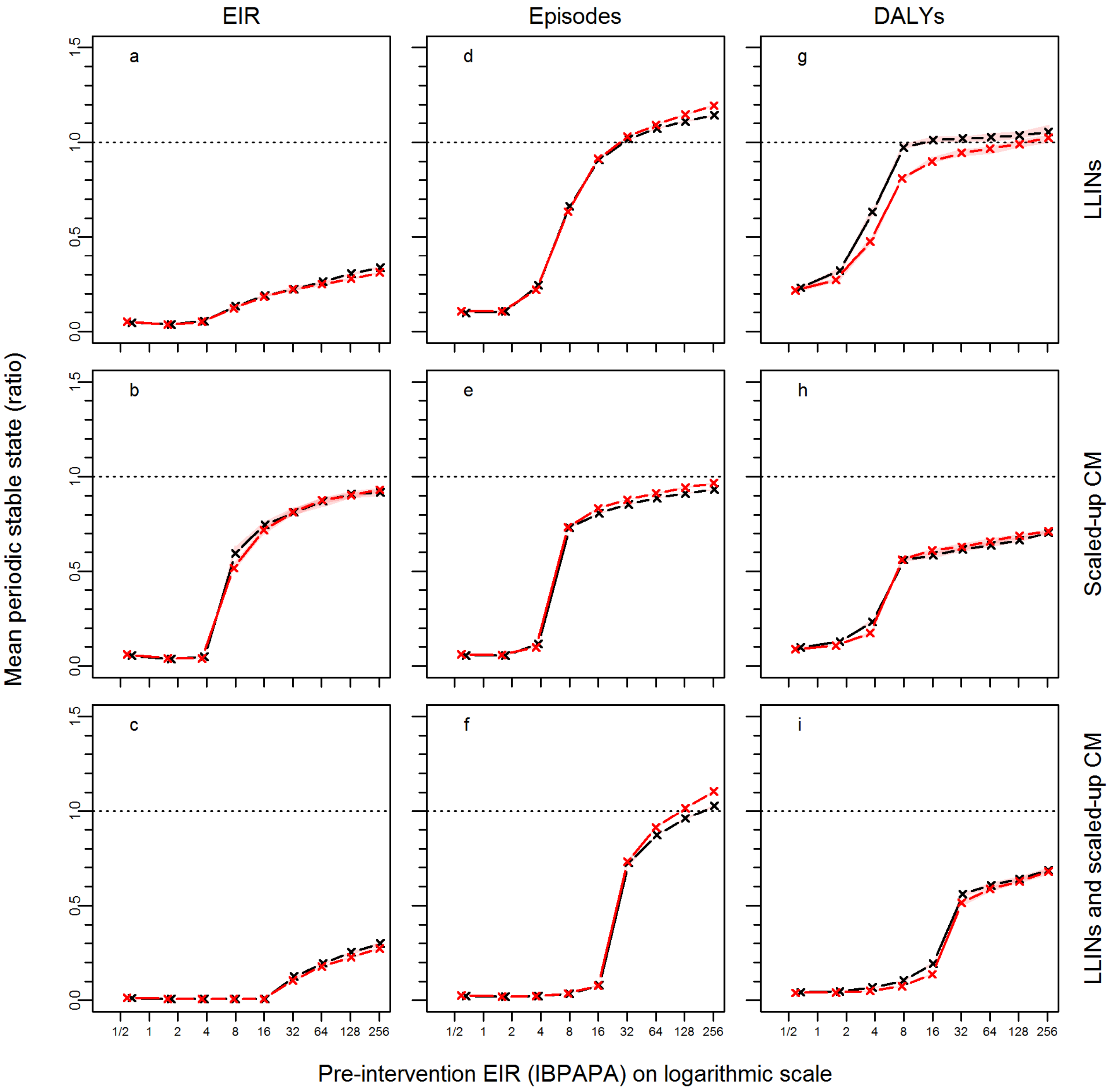

Figure S7.3 Impact of LLINs, CM or both on EIR, episodes and DALYs at periodic stable state. Ratios of results for intervention scenarios (a, d and g: long lasting insecticidal nets (LLINs); b, e and h scaled up case management (CM); and c, f and i both LLINs and scaled-up CM) and non-intervention scenarios (low CM only) calculated for means over the last 60 years of individual runs of 125 years, with 10 unique seeds per input EIR and model variant combination, for outcomes a, b and c: entomological inoculation rate (EIR): d, e and f: episodes; and g, h and i: disability adjusted life years (DALYs). Lines connect median values of groups of the ten seeds with the same input EIR and model variant. Black lines and dots represent model variant R1134 with the data of Marsh and Snow (1999) included in the fitting objectives. Red lines and dots represent model variant R3300, which is has exactly the same specifications as variant R1134 except that the data of Marsh and Snow (1999) were excluded from the fitting objectives. Red polygons show ranges.

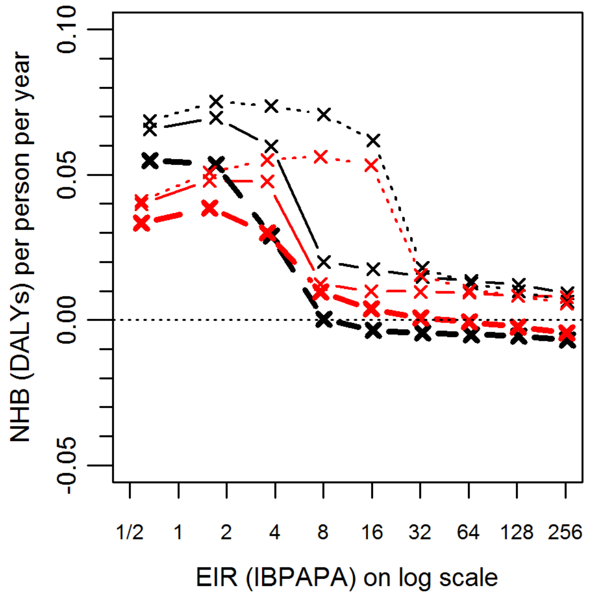

Figure S7.4 Mean NHBs of LLINs and CM at periodic stable state. Data are averaged over the last 60 years of the 125 year simulation period, where the net health benefits (NHB) are in a periodic stable state. NHB are calculated as compared to scenarios with a low baseline case management (CM) of 9% reported treatment of recalled fevers with an effective antimalarial drug in DHS type surveys. The thick solid lines show the effect of only distributing LLINs. The thin solid lines show the effect of only scaling up CM to 80% reported treatment of recalled fevers. The dotted lines show the effect of both distributing LLINs and scaling up CM to 80%. Black lines and crosses represent model variant R1134 with the data of Marsh and Snow 1999 included in the fitting objectives. Red lines and crosses represent model variant R3300, which is has exactly the same specifications as variant R1134 except that the data of Marsh and Snow (1999) were excluded from the fitting objectives.
