# Supplementary material for: Repeated mass distributions and continuous distribution of long-lasting insecticidal nets: modelling sustainability of health benefits from mosquito nets, depending on case management
Source: Malar J. 2013 Nov 7;12:401. doi: 10.1186/1475-2875-12-401 (PMC4228503; doi:10.1186/1475-2875-12-401)
Supplement: Additional file 8 — Sensitivity analysis. [file 1475-2875-12-401-S8.pdf]

Additional file 8: Sensitivity analysis

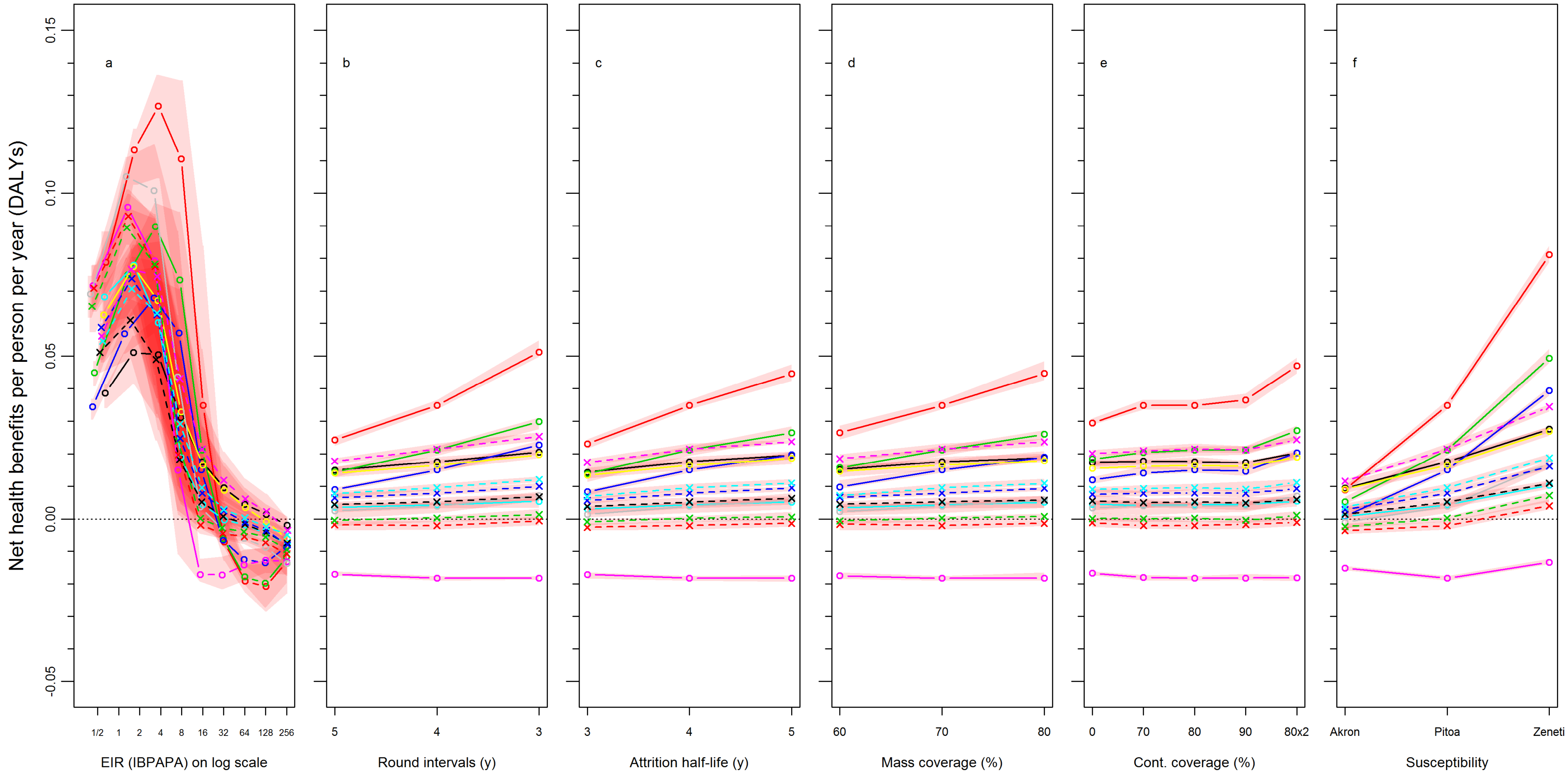

Figure S8.1 Sensitivity of NHB of LLINs to model parameters (Pre-intervention EIR of 16 IBPAPA). Plotted data are the median over 10 runs with unique seeds of the average NHB over the last 60 years of individual runs of 125 years **a**) varying the pre-intervention EIR; **b**) varying the susceptibility profile of the mosquito population; **c**) varying attrition half-life of the LLINs; **d**) varying round intervals; **e**) varying the population covered each mass distribution; and **f**) varying the coverage of continuous distribution to infants (and associated users). In panel **f**, '80x2' indicates a situation where 80% of new-borns received an LLIN during birth (*e.g.* through ANC), and also 80% received an LLIN shortly after birth (*e.g.* through EPI). If one LLIN was received per new-born, this was allocated to be shared between the new born and an adult in the fertile age range, and if two were received, the extra net was allocated to two people irrespective of age. Model variants are colour coded as described in the legend of Figure 5. The red shading indicated the range over the 10 runs with unique seeds.

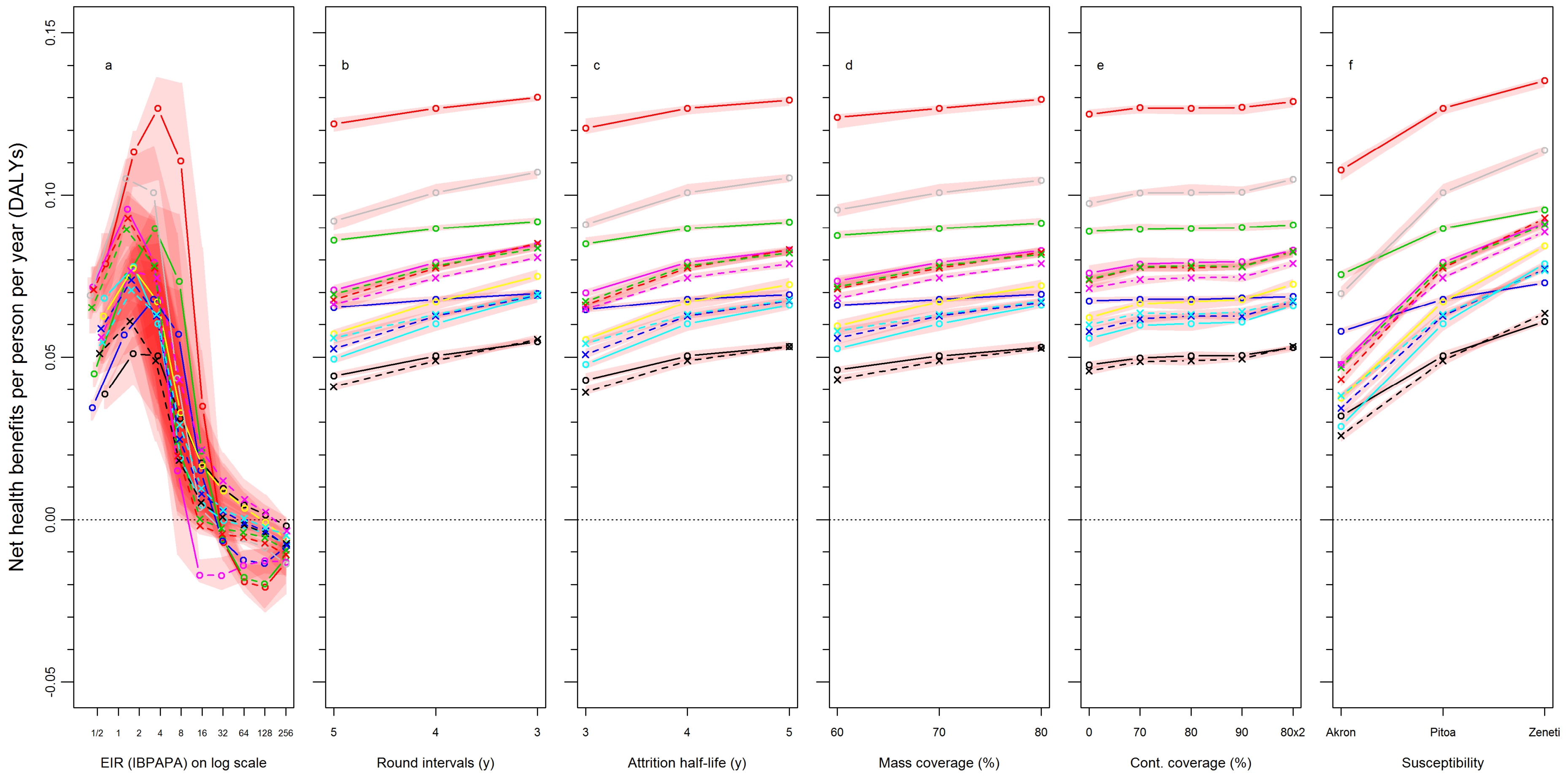

Figure S8.2 - Sensitivity of NHB of LLINs to model parameters (Pre-intervention EIR of 4 IBPAPA). See legend of Figure S8.1.
