# Supplementary material for: Repeated mass distributions and continuous distribution of long-lasting insecticidal nets: modelling sustainability of health benefits from mosquito nets, depending on case management
Source: Malar J. 2013 Nov 7;12:401. doi: 10.1186/1475-2875-12-401 (PMC4228503; doi:10.1186/1475-2875-12-401)
Supplement: Additional file 9 — Impact of LLIN distributions on EIR. [file 1475-2875-12-401-S9.pdf]

Additional file 9: Figure S9.1 Impact of LLIN distributions on EIR.

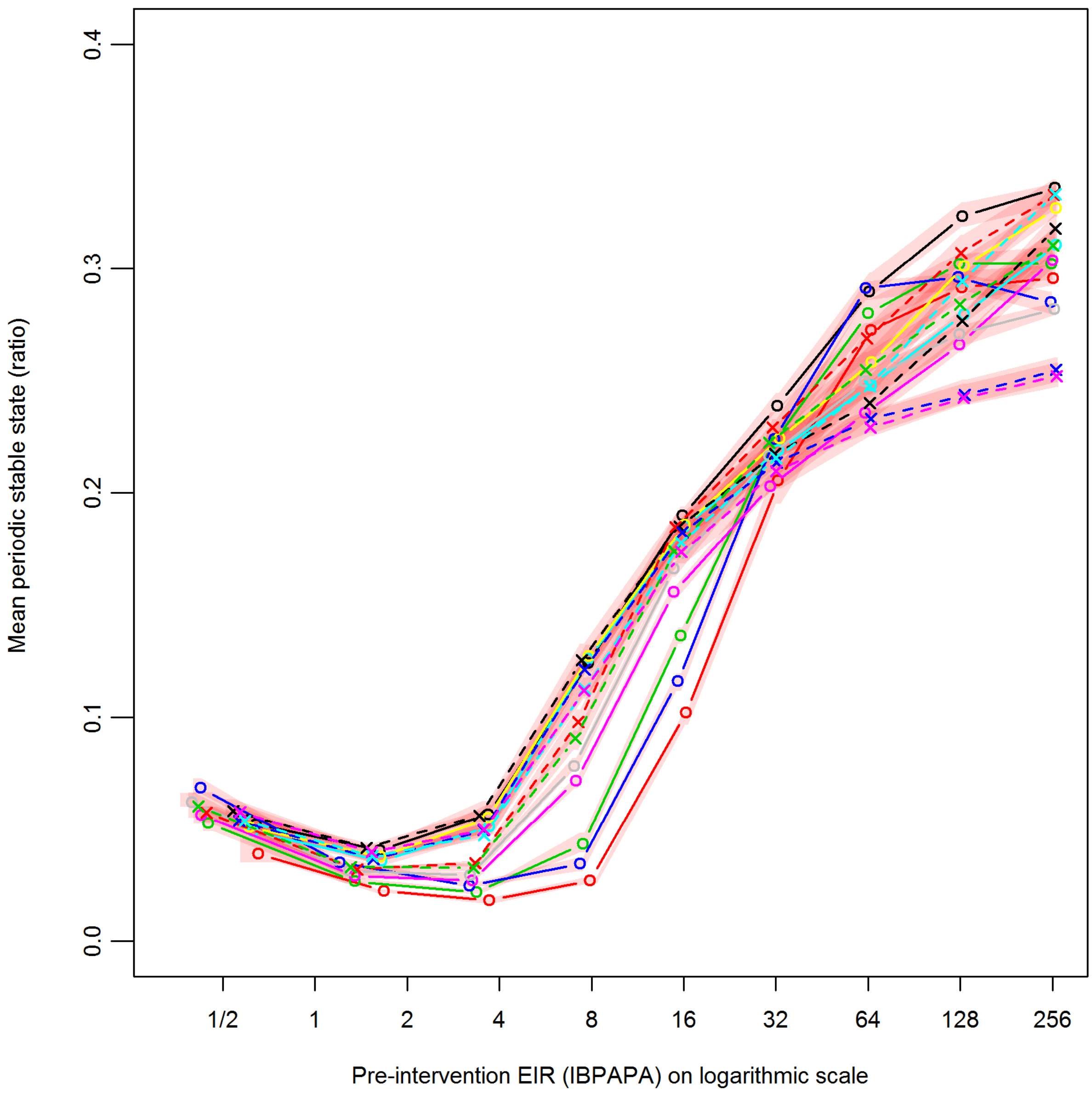

Figure S9.1 Impact of LLIN distributions on EIR (Figure 5a on different vertical scale). Ratios of results for intervention scenarios with long lasting insecticidal nets (LLINs) at low case management (CM) and non-intervention scenarios (low CM only) calculated for means over the last 60 years of individual runs of 125 years, with 10 unique seeds per input EIR and model variant combination, for entomological inoculation rate (EIR): Lines connect median values of groups of the ten seeds with the same input EIR and model variant. Model variants [17]: R0001 = solid black lines and circles; R0063 = solid red lines and circles; R0065 = solid lime green lines and circles; R0068 = solid blue lines and circles; R0111 = solid cyan lines and circles; R0115 = solid magenta lines and circles; R0121 = solid yellow lines and circles; R0125 = solid grey lines and circles; R0131 = dashed black lines and crosses; R0132 = dashed red lines and crosses; R0133 = dashed lime green lines and crosses; R0670 = dashed blue lines and crosses; R0674 = dashed cyan lines and crosses; R0678 = dashed magenta lines and crosses. Red polygons show ranges.
