# Supplementary material for: Repeated mass distributions and continuous distribution of long-lasting insecticidal nets: modelling sustainability of health benefits from mosquito nets, depending on case management
Source: Malar J. 2013 Nov 7;12:401. doi: 10.1186/1475-2875-12-401 (PMC4228503; doi:10.1186/1475-2875-12-401)
Supplement: Additional file 10 — Impact of halting LLIN distribution in scenarios with high CM coverage relative to continued high CM coverage. [file 1475-2875-12-401-S10.pdf]

Additional file 10: Impact of halting LLIN distribution in scenarios with high CM coverage relative to continued high CM coverage

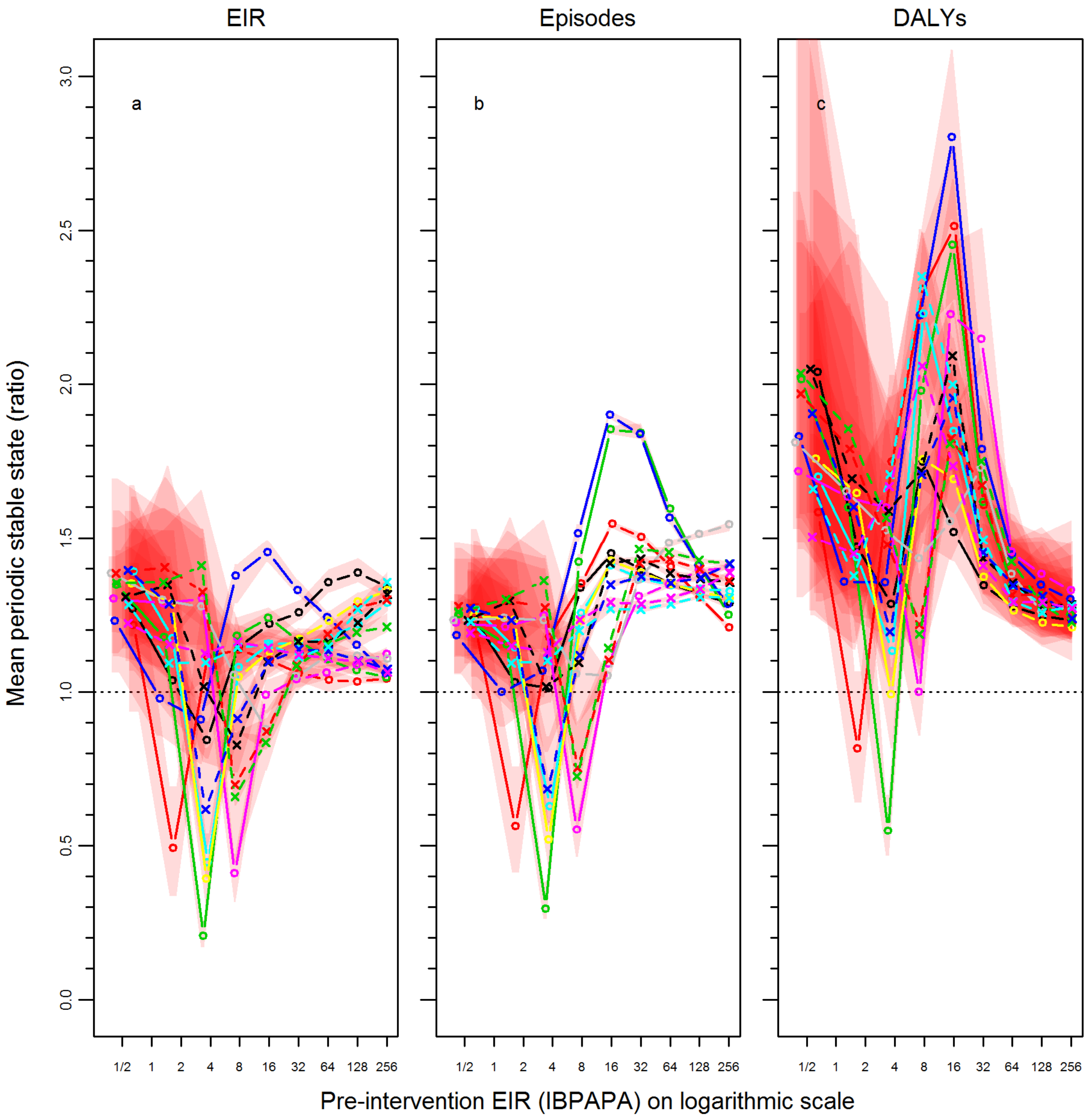

Figure S10.1 Maximum rebound after abruptly halting LLIN distributions, depending pre-intervention EIR, for high CM coverage, relative to scenarios with (continued) high CM coverage. Legend: Maxima in the ratios of results after halting LLIN distribution after eight rounds calculated for means over the last 60 years of individual runs of 125 years, with 10 unique seeds per input EIR and model variant combination, for outcomes **a:** entomological inoculation rate (EIR): **b**: episodes; and **c**: disability adjusted life years (DALYs). Lines connect median values of groups of the ten seeds with the same input EIR and model variant. Model variants [17]: R0001 = solid black lines and circles; R0063 = solid red lines and circles; R0065 = solid lime green lines and circles; R0068 = solid blue lines and circles; R0111 = solid cyan lines and circles; R0115 = solid magenta lines and circles; R0121 = solid yellow lines and circles; R0125 = solid grey lines and circles; R0131 = dashed black lines and crosses; R0132 = dashed red lines and crosses; R0133 = dashed lime green lines and crosses; R0670 = dashed blue lines and crosses; R0674 = dashed cyan lines and crosses; R0678 = dashed magenta lines and crosses. Red polygons show ranges. Note that if the rebound maximum is shown is below 1.0, it is possible that the maximum is not reached within 23 years post halting of LLIN distribution, and it is likely that in that case, the maximum is not larger than 1.0.
