# Supplementary material for: Repeated mass distributions and continuous distribution of long-lasting insecticidal nets: modelling sustainability of health benefits from mosquito nets, depending on case management
Source: Malar J. 2013 Nov 7;12:401. doi: 10.1186/1475-2875-12-401 (PMC4228503; doi:10.1186/1475-2875-12-401)
Supplement: Additional file 11 — Effect of imported infections. [file 1475-2875-12-401-S11.pdf]

Additional file 11: Effect of imported infections

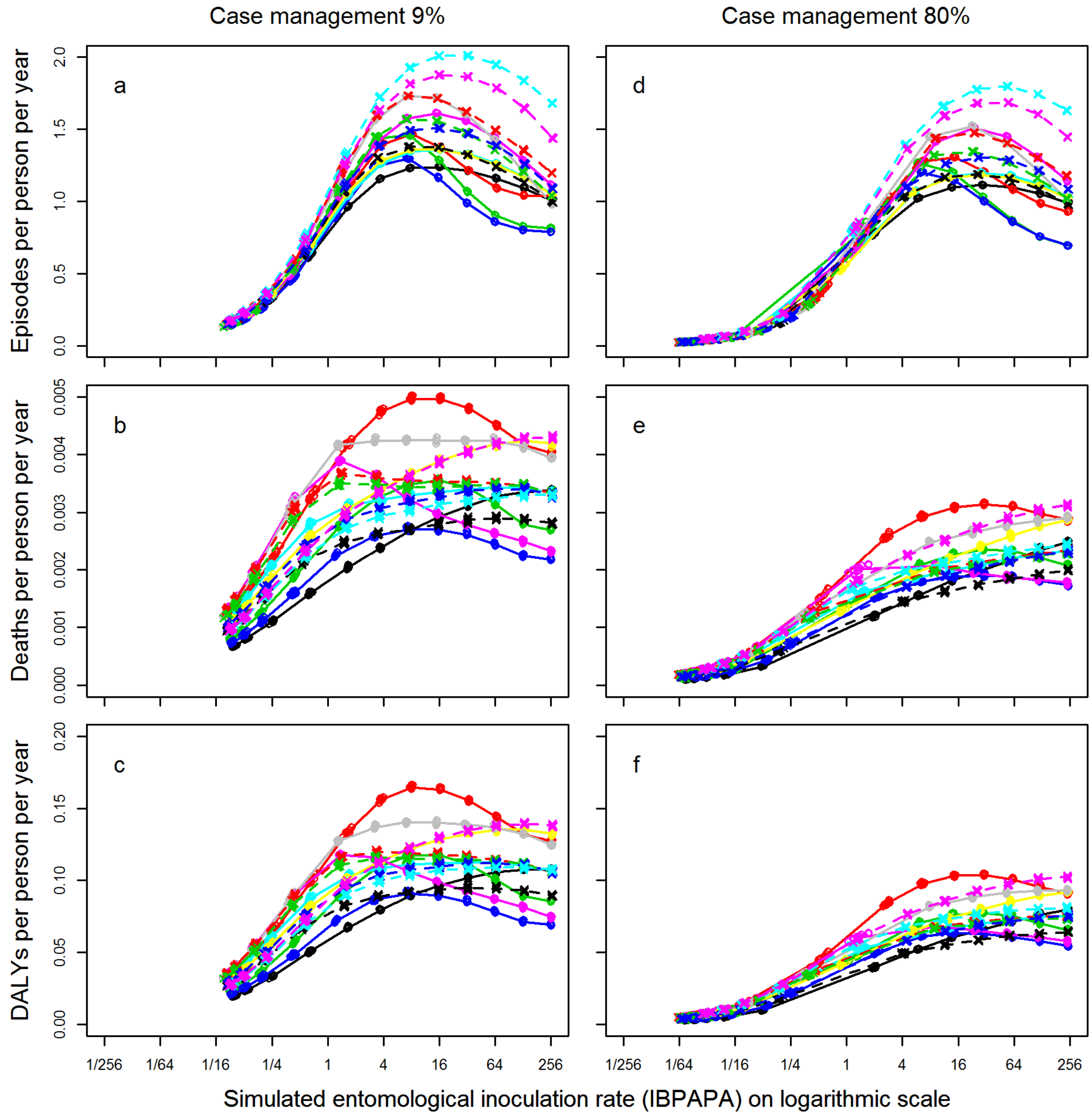

Figure S11.1 Episodes, deaths, and DALYs depending on transmission and CM without LLINs. Averages for the last 60 years of individual runs of 125 years, with 10 unique seeds per input EIR and model variant combination, with a & d) episodes per person per year b & e) direct and indirect deaths due to malaria, and c & f) disability adjusted life years (DALYs). Lines connect median values of groups of the ten seeds with the same input EIR and model variant. See the legend of Figure 5 for model variant colour coding. This figure is identical to Figure 6 in the main manuscript, except that the horizontal axis extends to 1/256 to allow visual comparison with Figure S11.2. In these scenarios, infections were imported into the population at a rate of 10 per 1000 population per year.

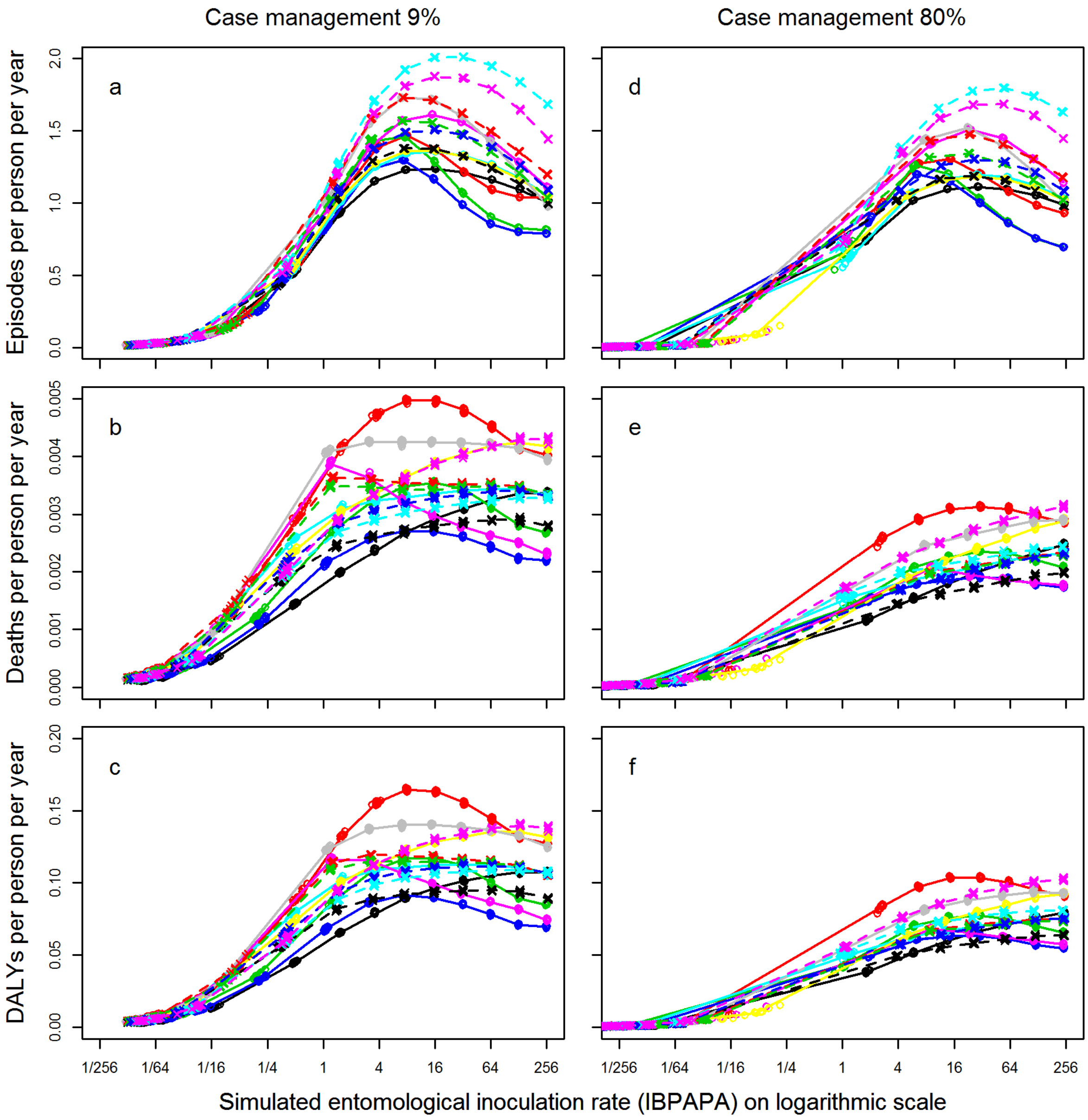

Figure 11.2 Episodes, deaths, and DALYs depending on transmission and CM without LLINs, with low infection importation rate. In these scenarios, infections were imported into the population at a rate of 1 per 1000 population per year. See further legend of Figure S11.1.
